# A-to-I RNA editing profiles and distinct immune responses in myelin oligodendrocyte glycoprotein antibody-positive optic neuritis

**DOI:** 10.3389/fimmu.2026.1946792

**Published:** 2026-09-03

**Authors:** Xueqing Cui, Jia-Qi Pan, Jianan Ding, Xuchu Fu, Fanli Cheng, Yutong Zhang, Jian-Huan Chen, Wenjun Zou

**Affiliations:** 1 Department of Ophthalmology, Jiangnan University Medical Center (JUMC), Wuxi, Jiangsu, China; 2 Department of Ophthalmology, Wuxi No. 2 People’s Hospital, Wuxi, Jiangsu, China; 3 Laboratory of Genomic and Precision Medicine, Wuxi School of Medicine, Jiangnan University, Wuxi, Jiangsu, China

**Keywords:** A-to-I RNA editing, gene expression, immune response, inflammation, MOG antibody-positive optic neuritis

## Abstract

**Introduction:**

Myelin oligodendrocyte glycoprotein antibody-positive optic neuritis (MOG-ON) is an autoimmune inflammatory demyelinating disorder of the optic nerve that often results in severe visual impairment and recurrent attacks. Although immune dysregulation is recognized as a central feature of MOG-ON, the contribution of post-transcriptional regulatory mechanisms, particularly adenosine-to-inosine (A-to-I) RNA editing, to disease pathogenesis and visual outcomes remains poorly understood. In this study, we characterized A-to-I RNA editing in MOG-ON, investigated molecular signatures and immune responses across the acute and remission phases, and explored candidate genes and associations with visual outcomes.

**Methods:**

A total of 14 peripheral blood samples were collected from patients with MOG-ON (9 in the acute phase and 5 in remission) and from 7 healthy controls. The A-to-I editome was characterized using RNA editing profiling, and stage-associated molecular signatures and immune responses were investigated using transcriptomic sequencing. In addition, functional enrichment analysis, immune pathway profiling, and estimated immune-cell proportions analysis were performed.

**Results:**

MOG-ON exhibited extensive A-to-I RNA editing remodeling, with 240 candidate differentially edited sites across 135 genes, predominantly enriched in neutrophil-mediated immunity and T-cell differentiation. Transcriptomic analysis revealed distinct stage-associated molecular programs: immunoglobulin production and B-cell receptor signaling were enriched during the acute phase, whereas cell cycle regulation was predominant during remission. CD74 and CDK1 were identified as candidate hub genes in the acute and remission phases, respectively. Furthermore, exploratory analyses suggested nominal associations of acute-phase BCVA with proportions of regulatory T cells and of CD74 expression with estimated neutrophil and monocyte proportions.

**Discussion:**

Collectively, these findings characterize RNA-editing alterations and stage-associated immune-transcriptomic features in MOG-ON and provide hypothesis-generating molecular candidates for further investigation.

## 1 Introduction

Myelin oligodendrocyte glycoprotein antibody–positive optic neuritis (MOG-ON) is the most common and clinically characteristic phenotype of myelin oligodendrocyte glycoprotein antibody–associated disease, an autoimmune inflammatory demyelinating disorder distinct from multiple sclerosis and neuromyelitis optica spectrum disorder (1, 2). The clinical characteristics of MOG-ON include bilateral ocular involvement, severe optic disc swelling, and longitudinally extensive inflammatory lesions with optic perineuritis, often resulting in recurrent visual impairment and irreversible visual dysfunction (3–5). However, the pathogenesis of MOG-ON is yet to be elucidated. Further characterization of the molecular and immune mechanisms of MOG-ON may help identify candidate biomarkers associated with disease activity and clinical outcomes (6, 7).

Transcriptomic sequencing has been extensively employed to determine the molecular mechanisms underlying neurological diseases. Nonetheless, as a post-transcriptional epigenetic modification, the functional characteristics and molecular landscape of adenosine-to-inosine (A-to-I) RNA editing in MOG-ON have not been fully clarified (8–12). To date, mechanistic studies of MOG-ON have largely focused on antibody-mediated immune activation and complement-dependent cytotoxicity, demonstrating that MOG antibodies can drive optic nerve demyelination and inflammatory cell infiltration. Nevertheless, stage-associated immune and molecular features of MOG-ON remain insufficiently characterized (11, 13, 14). Our previous study revealed distinct immune mechanisms between AQP4 antibody–positive optic neuritis (AQP4-ON) and MOG-ON and identified the translocator protein (TSPO) as a candidate molecule associated with AQP4-ON (15, 16). Transcriptomic profiling of peripheral blood from patients with AQP4-ON during the acute and remission phases further identified *TSPO* and *CDK1* as candidate hub genes associated with the acute and remission phases, respectively (17). Building on these preliminary findings, patients with MOG-ON were enrolled in this study; their peripheral whole-blood samples were collected during acute and remission periods, and RNA sequencing was performed on these samples. The aim was to characterize the landscape of A-to-I RNA editing in MOG-ON, reveal phase-specific immune regulatory networks associated with different disease stages, and examine the link between key molecular signatures and visual prognosis.

## 2 Materials and methods

### 2.1 Patients and samples

From January 2022 to September 2024, 12 patients with MOG-ON were recruited from the Department of Ophthalmology, Wuxi No. 2 People’s Hospital. Peripheral blood samples (9 from the acute phase and 5 from the remission phase) were obtained from the patients. In addition, 7 healthy volunteers were enrolled as controls. The diagnosis of optic neuritis was made according to the Optic Neuritis Treatment Trial (ONTT) criteria. MOG-ON was defined by a clinical diagnosis of optic neuritis together with serum MOG-IgG positivity. If the MOG-IgG result was only low-positive, at least one supportive clinical or MRI feature, such as bilateral optic neuritis, involvement of over half of the optic nerve, optic nerve sheath enhancement, or optic disc edema, together with negative AQP4-IgG, was considered to reduce the risk of misdiagnosis. Serum MOG-IgG was detected by indirect immunofluorescence testing (IIFT) using a cell-based assay (CBA). Patients with neurological diseases, such as encephalitis and myelitis, and eye diseases (such as glaucoma, uveitis, and ischemic optic neuropathy (but not cataracts) were excluded. Acute-phase samples were collected before corticosteroid treatment, whereas remission-phase samples were collected during outpatient follow-up 3–6 months after the attack. Peripheral blood samples were collected from two patients at both the acute and remission phases, with sampling intervals of 6 months for Patient 2 and 4 months for Patient 9. We have defined the attack state as the period within 1 month of the current optic neuritis attack before initiating the corticosteroid treatment, and the remission state as the stable phase at least 3 months following the initial attack with partial or complete recovery of visual symptoms. Best-corrected visual acuity (BCVA) was converted to logMAR units for statistical analysis. For visual-outcome analyses, each patient was treated as one independent observation. In patients with unilateral optic neuritis, BCVA of the affected eye was used. In patients with simultaneous bilateral involvement, the eye with worse BCVA at baseline was selected as the study eye, and the same eye was used for subsequent visual-outcome analyses. The study was approved by the Ethics Committee of the Wuxi No. 2 People’s Hospital (No. 2021Y-33). This study was conducted in accordance with the Declaration of Helsinki, and all participants provided written informed consent. (**Table 1**).

**Table 1:** Treatment and prognosis of the study subjects.

Patient ID	Sample	Sex	Age	Affected eyes	BCVA at attendance	Last follow-up BCVA	MOG-IgG titer	Treatment status at time of sampling
Acute stage								
Patient 1	A1	Male	18	Left	0.4	0	1:10	–
Patient 2	A2	Female	33	Left	0.5	0	1:10	–
Patient 3	A3	Female	57	Left	1.1	0.7	1:10	–
Patient 4	A4	Female	24	Right	0.2	0	1:100	–
Patient 5	A5	Female	39	Right	2.5	0	1:10	–
Patient 6	A6	Male	63	Right	2	3.0	1:10	–
Patient 7	A7	Female	72	Left	2	0.7	1:100	–
Patient 8	A8	Male	35	Right	2.5	0.9	1:32	–
Patient 9	A9	Male	61	Left	0.4	0.3	1:10	–
Remission stage								
Patient 9	R1	Male	61	Left	0.4	0.3	negative	IVMP and oral prednisolone
Patient 10	R2	Female	40	Left	2.5	0.1	1:10	IVMP, rituximab and oral prednisolone
Patient 11	R3	Female	15	Left	0	0	1:10	–
Patient 12	R4	Female	26	Right	1.3	0.5	negative	IVMP and oral prednisolone
Patient 2	R5	Female	33	Left	0.5	0	1:10	IVMP and oral prednisolone

Age, years at diagnosis; IVMP, intravenous pulses of methylprednisolone. BCVA, Snellen visual acuity was converted to logarithm of the minimum angle of resolution (logMAR). Counting fingers vision was converted to a value of 2.0 logMAR, hand motion vision was converted to a value of 2.5 logMAR, light perception vision was converted to a value of 3.0 logMAR and no light perception vision was converted to a value of 3.5 logMAR.

### 2.2 RNA extraction and library construction

Peripheral venous whole blood (2 mL) was collected from patients with MOG-ON and healthy controls into vacuum tubes containing EDTA as an anticoagulant. After collection, 4 mL of TRIzol reagent (Beyotime, Nantong, China) was added to each sample and stored at −80 °C for subsequent RNA extraction. Total RNA was extracted using TRIzol reagent according to the manufacturer’s instructions. RNA integrity was assessed using an Agilent 2100 Bioanalyzer (Agilent Technologies Inc., Santa Clara, CA, USA). Qualified RNA was reverse-transcribed into complementary DNA (cDNA). The purified double-stranded cDNA was end-repaired, A-tailed, and ligated to sequencing adapters. AMPure XP beads (Beckman Coulter Inc., Brea, CA, USA) were used for size selection of cDNA fragments of approximately 250–300 bp. Following PCR amplification, the amplified products were purified again using AMPure XP beads. Library quality was assessed using a Qubit 2.0 Fluorometer and an Agilent 2100 Bioanalyzer, and the effective library concentration was quantified by qPCR. Qualified libraries were sequenced on the Illumina NovaSeq 6000 platform (Illumina Inc., San Diego, CA, USA).

### 2.3 RNA-seq and differentially expressed gene analysis

After preprocessing the obtained data, including quality control, removing low-quality sequences, and trimming the adapter sequences, clean reads were aligned to a reference genome or transcriptome to obtain the expression counts for each gene. The raw data have been submitted to SRA and GEO database (accession numbers: PRJNA1497092, GSE339140). Clean reads were aligned to the reference genome using HISAT2 (version 2.0.5). Gene-level raw read counts were generated using featureCounts (version 1.5.0-P3). Differential expression analysis was performed using DESeq2 (version 1.20.0). Genes with a cumulative read count greater than 1 across all samples were retained for analysis. Raw read counts were normalized using the normalization procedure implemented in DESeq2 to account for differences in sequencing depth among samples. Differential expression was assessed using a negative binomial model. The design matrix included disease group as the sole explanatory variable, with no additional covariates incorporated into the model. Given the exploratory nature of this study, genes with a nominal P value ≤0.05 were considered candidate differentially expressed genes (DEGs) for downstream exploratory analyses. P values were additionally adjusted for multiple testing using the Benjamini–Hochberg (BH) procedure and are reported in the **Supplementary Data**. All samples were processed in the same batch using an identical RNA extraction, library preparation, and sequencing workflow on the same sequencing platform, thereby minimizing potential technical batch effects.

### 2.4 Variant-calling, annotation, and filtering

VarScan (version 2.4.4) and the Ensembl Variant Effect Predictor (VEP) were used for the identification and annotation of A > G single nucleotide variations (SNVs) (18, 19). SNVs meeting specific criteria were selected, including a base quality of ≥25, total sequencing depth of ≥10, alternative allele depth of ≥2, and alternative allele frequency (AAF) of ≥1%. For A-to-I RNA editing analysis, only A-to-G SNVs on the coding strand were retained. SNVs were excluded if they were located in mitochondrial regions, homopolymer runs (≥5 nt), simple repeats, splice junctions (≤6 nt), indels (≤1 nt), or read ends (≤4%); variants annotated in dbSNP Build 142; or observed in more than 90% of samples with an AAF of either 100% or between 40% and 60%, unless they were annotated as known RNA-editing events in REDIportal V2.0 (20). Putative high-confidence A-to-G RNA-editing events were defined as variants detected at an editing level of ≥1% in at least two samples or annotated in REDIportal V2.0 (21, 22). Only putative high-confidence RNA-editing events were included in subsequent analyses. Intergroup differences in RNA-editing levels were assessed using a generalized linear model (GLM). Given the exploratory nature of the present study, RNA-editing sites with a nominal GLM P value <0.05 were considered candidate differentially edited sites and retained for downstream exploratory analyses. BH-adjusted P values were also calculated to account for multiple testing and are reported in the **Supplementary Table 1**.

### 2.5 Biological function analysis

Gene Ontology (GO) enrichment and Kyoto Encyclopedia of Genes and Genomes (KEGG) pathway enrichment analyses were performed on candidate DEGs and genes harboring candidate differentially edited sites using the clusterProfiler R package (version 3.8.1). GO terms and KEGG pathways with *P* < 0.05 were considered significantly enriched. Protein–protein interaction (PPI) networks for these gene sets were constructed using STRING and visualized in Cytoscape 3.9.1. Candidate hub genes were identified based on degree connectivity.

### 2.6 Peripheral immune-cell composition analysis

Cell-type identification by estimating relative subsets of RNA transcripts (CIBERSORTx) (https://cibersortx.stanford.edu/) is a method to characterize the cell composition of complex tissues from gene expression profiles (GEPs). CIBERSORTx was used to estimate the relative proportions of 22 immune-cell subsets from peripheral blood gene-expression profiles. Then, the Immune Cell Abundance Identifier (ImmuCellAI) gene set signature-based method (https://guolab.wchscu.cn/ImmuCellAI/) was used to estimate peripheral immune-cell proportions. CIBERSORTx was applied to the gene expression matrix, expressed as FPKM values, using the LM22 signature matrix to estimate the relative proportions of 22 peripheral immune cell subsets. The analysis was performed in relative mode with 100 permutations. Quantile normalization was disabled in accordance with the recommended settings for RNA-seq data, and no batch correction was applied. Sample-level deconvolution quality was evaluated using the empirical deconvolution P value, correlation coefficient, and root mean square error (RMSE) generated by CIBERSORTx (**Supplementary Table 4**). In parallel, ImmuCellAI was independently applied to estimate peripheral immune-cell proportions. To assess cross-method concordance, immune-cell populations with comparable definitions across the two algorithms were included in paired comparisons. Spearman correlation analyses were performed using matched estimates from all 21 samples. For NK cells, the resting and activated NK-cell fractions estimated by CIBERSORTx were summed before comparison with the corresponding NK-cell estimates generated by ImmuCellAI. P values from the cross-method correlation analyses were adjusted for multiple testing using the Benjamini–Hochberg procedure (**Supplementary Table 5**; **Supplementary Figure 1**).

### 2.7 Statistical analysis

Statistical procedures for the identification of candidate differentially edited RNA sites are described in Section 2.4. Statistical analyses of clinical data were performed using SPSS version 26.0 and GraphPad Prism version 9.0. Best-corrected visual acuity (BCVA) was converted to logarithm of the minimum angle of resolution (logMAR) units for statistical analysis. Counting fingers, hand motion, light perception, and no light perception were assigned logMAR values of 2.0, 2.5, 3.0, and 3.5, respectively. Continuous variables were compared using one-way analysis of variance or the Kruskal–Wallis test, as appropriate. Spearman’s rank correlation was used to evaluate associations between molecular or estimated immune-cell variables and visual outcomes, as well as correlations between RNA-editing levels and gene expression. Correlations between CD74 expression and estimated immune-cell proportions were performed at the sample level, with each peripheral blood sample treated as one observation. For correlation analyses, the exact number of observations and 95% confidence intervals for Spearman’s *r*s were reported. Where multiple correlations were tested, *P* values were adjusted using the Benjamini–Hochberg procedure. Complete results of the correlation analyses are provided in **Supplementary Table 6**. Data are presented as mean ± standard deviation or median, as appropriate. A two-sided *P* value <0.05 was considered statistically significant unless otherwise specified.

## 3 Results

### 3.1 Demographic and clinical features of patients

A total of 12 patients with MOG-ON and 7 HCs were recruited. The MOG-ON cohort comprised 9 samples in the acute phase (A) and 5 in the remission phase (R). Peripheral blood samples from two patients were collected in both phases. The mean age was 44.67 ± 19.05 years in Group A and 35.00 ± 17.22 years in Group R (*P* = 0.37). The female-to-male ratio was 5:4 in Group A and 4:1 in Group R (*P* = 0.58). The intraocular pressure was 15.04 ± 3.34 mmHg in Group A and 16.78 ± 3.12 mmHg in Group R (*P* = 0.33). At disease onset, the BCVA was 1.29 ± 0.96 logMAR in Group A and 0.85 ± 0.91 logMAR in Group R (*P* = 0.39). At the last follow-up, the BCVA was 0.62 ± 0.96 logMAR in Group A and 0.17 ± 0.20 logMAR in Group R (*P* = 0.50). The MOG-IgG titer was 32.44 ± 38.97 in Group A and 6.00 ± 5.48 in Group R, with a statistically significant difference between the two groups (*P* = 0.035). No significant differences were observed between the HCs and the patients in terms of age or sex ratio (*P* = 0.98 and *P* = 0.83, respectively; **Tables 1**, **2**).

**Table 2:** Characteristics of subjects with MOG-ON and healthy controls (HCs).

Characteristic	Acute (n=9)	Remission (n=5)	Total (n=12)	HC(n=7)	P^1^	P^2^
Age (years)	44.67 ± 19.05	35.00 ± 17.22	41.21 ± 18.37	41.42 ± 3.99	0.37	0.98
Sex (female/male)	5/4	4/1	2/1	5/2	0.58	0.83
IOP (mmHg)	15.04 ± 3.34	16.78 ± 3.12	–	–	0.33	–
BCVA at diagnosis	1.29 ± 0.96	0.85 ± 0.91	–	–	0.39	–
BCVA at the last follow-up	0.62 ± 0.96	0.17 ± 0.20	–	–	0.50	–
MOG antibody titers	32.44 ± 38.97	6.00 ± 5.48	–	–	0.035	–

Age, years at diagnosis; IOP, Intra-Ocular Pressure; BCVA, Snellen visual acuity was converted to logarithm of the minimum angle of resolution (logMAR). P^1^: Comparison between acute MOG-ON and remission MOG-ON. P^2^: Comparison between total MOG-ON and HCs. All statistical values are expressed as the mean ± standard deviation (SD), unless otherwise indicated.

### 3.2 A-to-I RNA editing in the epitranscriptome of patients with MOG-ON

**Figure 1:**
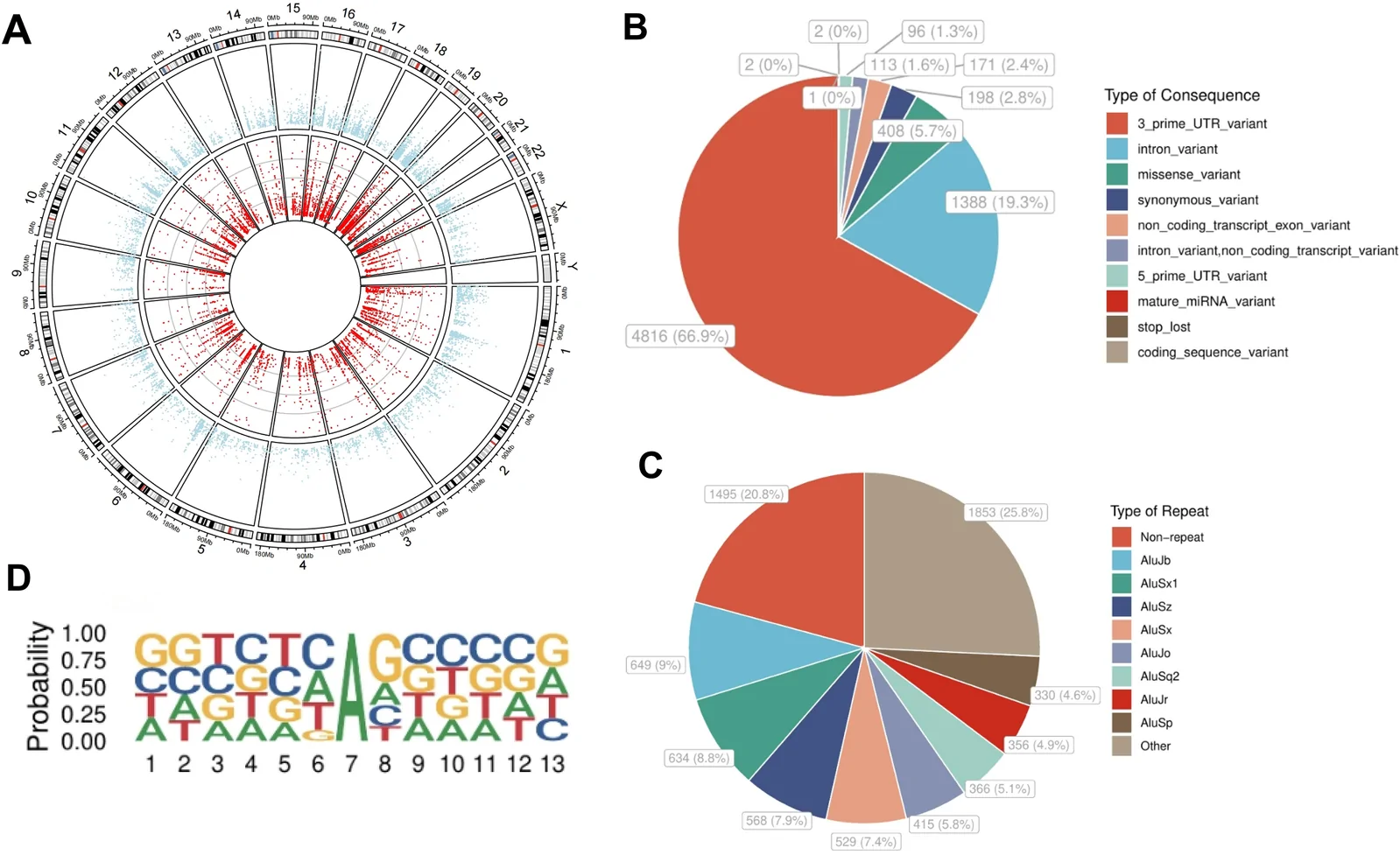
Landscape of putative high-confidence A-to-I RNA editing sites in peripheral blood transcriptomes. Samples included 14 MOG-ON samples (9 acute-phase and 5 remission-phase samples) and 7 HCs. **(A)** Dots in red denote the average RNA editing level of A-to-I RNA editing sites, and blue dots denote the average RNA expression of genes. **(B)** Functional categories of these A-to-I RNA editing sites. **(C)** The distribution of repetitive elements overlapping with the A-to-I RNA editing sites. **(D)** The motif of sequence context surrounding the A-to-I RNA editing sites. Six nucleotides upstream and downstream of the editing sites are shown.

In total, 7, 190 putative high-confidence A-to-I RNA editing events were identified in the RNA of 1, 208 genes across all samples (**Figure 1A**). These included 4, 816 (66.9%) 3′-untranslated region (UTR) sites, 1, 388 (19.3%) protein-coding intronic sites, 408 (5.7%) missense sites, 198 (2.8%) synonymous sites, 171 (2.4%) noncoding transcript exonic sites, 113 (1.6%) intronic sites within noncoding transcripts, and 96 (1.3%) 5′-UTR sites (**Figure 1B**). The repeats that overlapped most with these sites belonged to the Alu family (**Figure 1C**). Motif analysis of flanking sequences revealed that G was suppressed one base upstream of the editing sites but preferred one base downstream (**Figure 1D**).

### 3.3 A-to-I RNA editing signatures associated with MOG-ON

**Figure 2:**
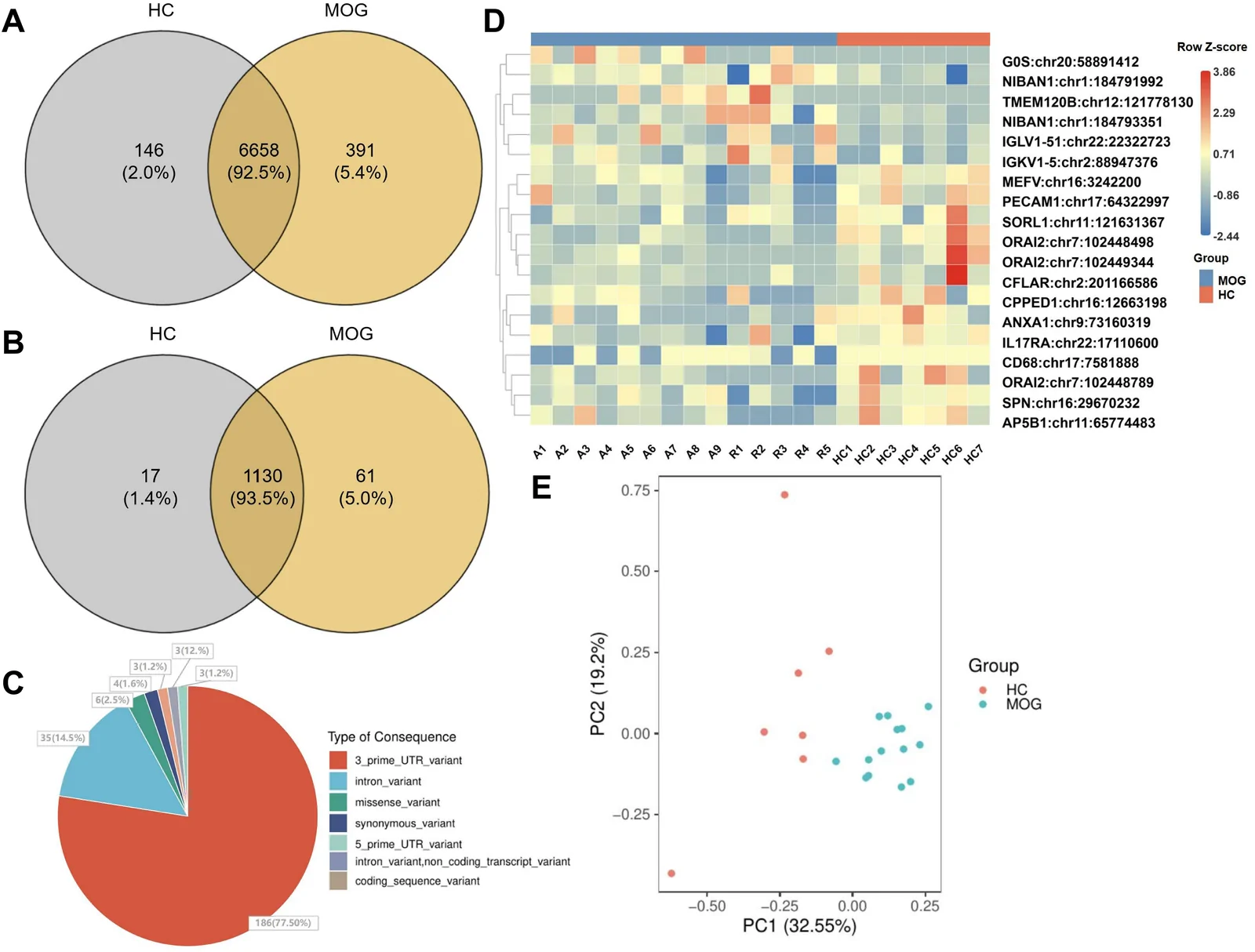
A-to-I RNA editing associated with MOG-ON. Samples included 14 MOG-ON samples and 7 HCs. **(A)** Venn plot of A-to-I RNA editing sites between MOG-ON and HC group. **(B)** Venn plot for comparison of A-to-I RNA edited genes among the groups. **(C)** Functional categories of candidate DRE sites. **(D)** Heatmap of 19 candidate DRE sites across individual samples. **(E)** PCA of the selected candidate DRE sites. Candidate DRE sites were identified by GLM at nominal P < 0.05, with BH-adjusted P values reported in **Supplementary Table 1**.

The comparison of A-to-I RNA editing sites and genes between MOG-ON and control groups is depicted in **Figure 2**. Of all putative high-confidence RNA editing sites, 6, 658 (92.5%) were common to both groups, whereas 146 (2.0%) and 391 (5.4%) were unique to controls and MOG, respectively (**Figure 2A**). Of the 1, 208 edited genes, 1, 130 (93.5%) were shared by both groups, whereas 17 (1.4%) and 61 (5.0%) were unique to controls and MOG, respectively (**Figure 2B**). The functional categories of all DRE sites are illustrated in **Figure 2C**, and the complete list of candidate differentially edited RNA sites is provided in **Supplementary Table 1**. To visualize RNA-editing patterns at the individual-sample level, 19 candidate DRE sites with sufficient editing-level data coverage were selected and displayed in a heatmap across acute-phase, remission-phase, and HC samples (**Figure 2D**). Principal component analysis (PCA) based on these sites revealed that the samples formed distinct clusters by group. The first and second principal components accounted for 32.55% and 19.2% of the total variance, respectively (**Figure 2E**). Of these DRE sites, six were missense, namely, p.K368R (c.1103A>G) in cerebral cavernous malformation 2 (*CCM2*), p.I274V (c.820A>G) in torsin-1B (*TOR1B*), p.K36R (c.107A>G) in immunoglobulin lambda variable 1-51 (*IGLV1-51*), p.T42A (c.124A>G) in immunoglobulin lambda variable 1-40 (*IGLV1-40*), p.K46R(c.137A>G) in immunoglobulin lambda joining 3 (*IGLJ3*) and p.T9A (c.25A>G) in immunoglobulin heavy joining 4 (*IGHJ4*). Of these, two missense sites, p.K36R (c.107A>G) and p.K46R (c.137A>G), demonstrated elevated RNA editing levels in the MOG-ON group, whereas the remaining four sites exhibited decreased editing. Although missense variants account for only a small fraction, they can affect protein structure and stability (23). These findings suggest characteristic alterations in peripheral blood RNA editing profiles among patients with MOG-ON.

### 3.4 A-to-I RNA editing–related functional enrichment in MOG-ON

**Figure 3:**
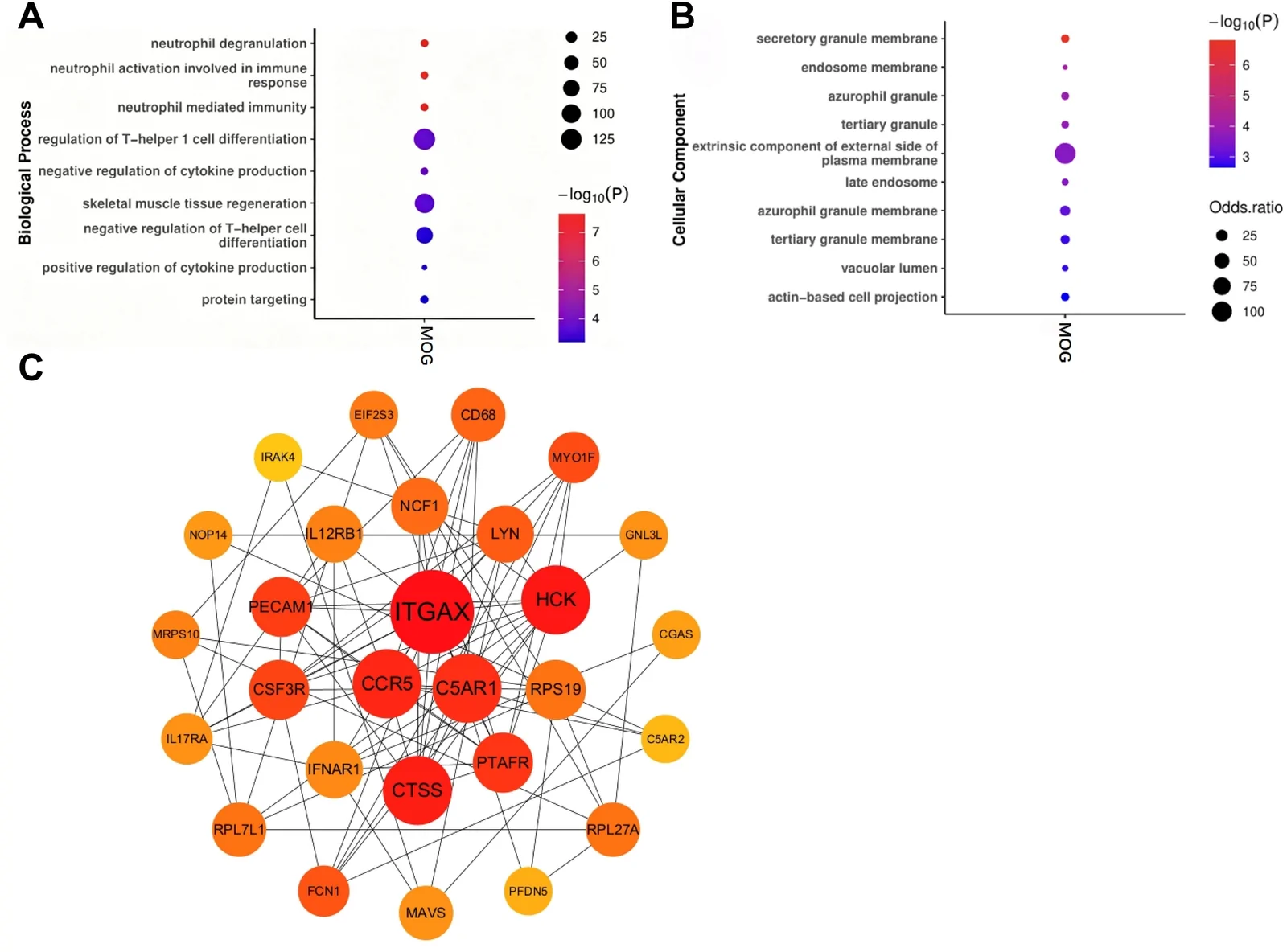
Functional enrichment and protein–protein interaction (PPI) network analyses of candidate differentially edited genes in MOG-ON. Samples included 14 MOG-ON samples and 7 HCs. **(A)** GO biological process (BP) enrichment. **(B)** GO cellular component (CC) enrichment. **(C)** PPI network analysis of candidate differentially edited genes. Candidate differentially edited genes were derived from the candidate DRE sites identified in **Figure 2**.

To assess the biological impact of A-to-I RNA editing in MOG-ON, enrichment analysis of candidate differentially edited genes that differed between the control and MOG-ON groups was performed. GO analysis revealed that neutrophil degranulation, neutrophil activation involved in immune responses, neutrophil-mediated immunity, regulation of T helper 1 cell differentiation, and negative regulation of cytokine production were the biological processes (BPs) enriched in MOG-ON (**Figure 3A**). Secretory granule membrane, endosome membrane, azurophil granule, and tertiary granule were the cellular components enriched in MOG-ON (**Figure 3B**). Compared with the control group, the MOG-ON group showed widespread alterations in the editome, including 240 candidate differentially edited sites and 135 candidate differentially edited genes, with candidate genes related to immune and inflammatory responses, such as *ITGAX* and *C5AR1* (**Figure 3C**).

### 3.5 Summary of RNA-seq data and disparities in gene expression patterns

**Figure 4:**
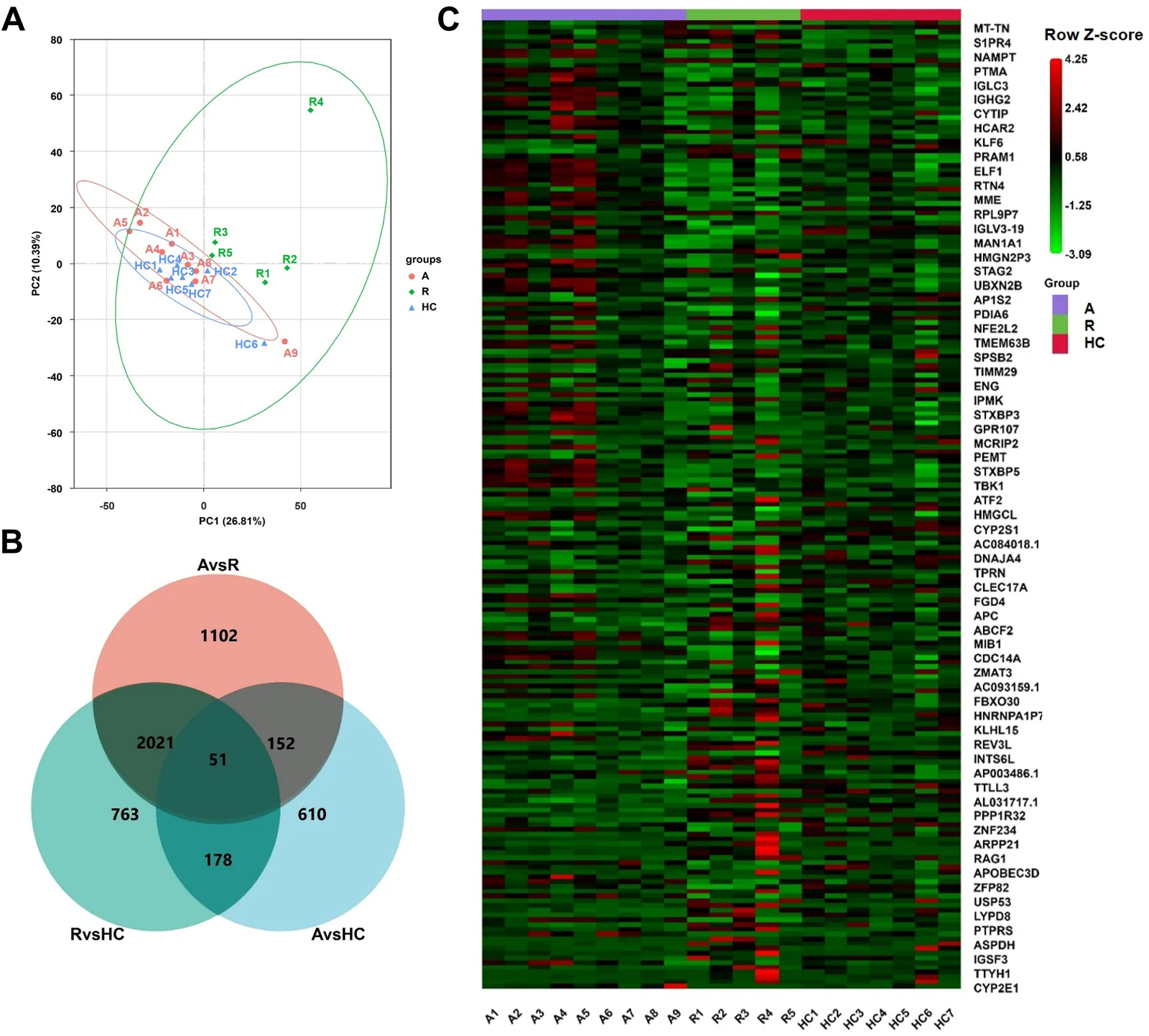
Differential gene-expression profiling and clustering analysis across acute-phase, remission-phase, and HC groups. Samples included A group (n = 9), R group (n = 5), and HC group (n = 7). **(A)** Principal component analysis (PCA) of gene-expression profiles. **(B)** Venn plot showing overlapping candidate DEGs among the three pairwise comparisons. **(C)** Heatmap of candidate DEGs across individual samples. Expression levels were standardized by row Z-score; red indicates higher expression and green indicates lower expression.

Gene expression matrices from patients with MOG-ON and HCs were standardized, and a PCA plot was generated to identify outlier samples and evaluate sample reproducibility (**Figure 4A**). PCA revealed partial separation among the three groups, although substantial overlap was observed, with PC1 and PC2 accounting for 26.81% and 10.39% of the total variance, respectively. The Venn plot showed that 51 candidate DEGs were shared across all three pairwise comparisons, 610 candidate DEGs were unique to Group A, and 763 candidate DEGs were unique to Group R (**Figure 4B**). Furthermore, the heatmap illustrated the individual-sample expression patterns of the identified genes across the three groups (**Figure 4C**).

**Figure 5:**
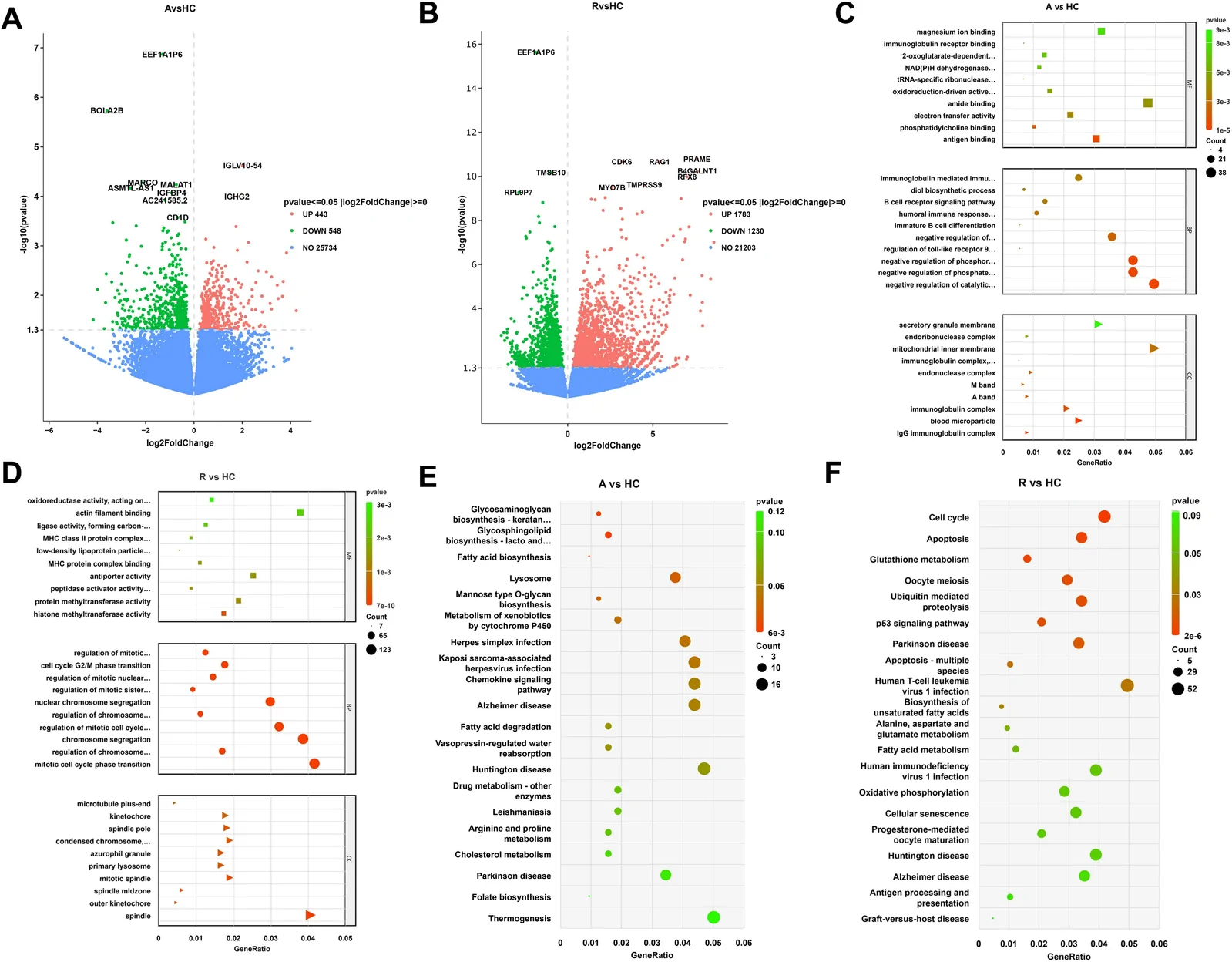
Differential gene-expression profiling and functional enrichment analysis in the acute and remission phases of MOG-ON. Samples included A group (n = 9), R group (n = 5), and HC group (n = 7). **(A)** Volcano diagram of the candidate DEGs in group A and the HC group. **(B)** Volcano diagram of the candidate DEGs in group R and the HC group. **(C)** GO term enrichment analysis in the A group and HC group. **(D)** GO term enrichment analysis in group R and the HC group. **(E)** KEGG enrichment analysis in group A and the HC group. **(F)** KEGG enrichment analysis in group R and the HC group. GO and KEGG enrichment was considered significant at *P* < 0.05.

Specifically, 991 candidate DEGs were identified between Group A and HCs, of which 548 were downregulated and 443 were upregulated. For all candidate DEGs, see **Supplementary Table 2**. In Group A, the expressions of *IGLV10–54* and *IGHG2* were significantly increased, whereas those of *EEF1A1P6*, *BOLA2B*, *MARCO*, *MALAT1*, *ASMTL-AS1*, *IGFBP4*, *AC241585.2*, and *CD1D* were significantly decreased, compared with HCs (**Figure 5A**). In total, 3, 013 candidate DEGs were detected between Group R and HCs, of which 1, 783 were upregulated, and 1, 230 were downregulated. In Group R, the expressions of *CDK6*, *RAG1*, *PRAME*, *MYO7B*, *B4GALNT1*, *RFX8*, and *TMPRSS9* were significantly increased, whereas those of *EEF1A1P6*, *TMSB10*, and *RPL9P7* were significantly decreased, compared with the HCs (**Figure 5B**).

For the biological functional analysis of candidate DEGs, GO term and KEGG pathway enrichment analyses were performed to identify significantly enriched BP terms and KEGG pathways. In the GO enrichment analysis of the acute phase (Group A vs. HCs), terms including “antigen binding, “ “immunoglobulin receptor binding, “ and “phosphatidylcholine binding” were significantly enriched in the molecular function (MF) category. Moreover, terms such as “immunoglobulin-mediated immune response, “ “B-cell receptor signaling pathway, “ and “negative regulation of phosphorylation” were significantly enriched in the BP category (**Figure 5C**). By contrast, in the GO enrichment analysis of the remission phase (Group R vs. HCs), terms such as “histone methyltransferase activity, “ “MHC class II protein complex binding, “ and “actin filament binding” were significantly enriched in the MF category. In addition, terms including “regulation of chromosome segregation, “ “regulation of mitotic cell cycle phase transition, “ and “cell cycle G2/M phase transition” were significantly enriched in the BP category (**Figure 5D**). In the KEGG pathway enrichment analysis of the acute phase (Group A vs. HCs), “glycosphingolipid biosynthesis-lacto and neolacto series, “ “lysosome, “ and “chemokine signaling pathway” were significantly enriched among the candidate DEGs (**Figure 5E**). Conversely, in the KEGG pathway enrichment analysis of the remission phase (Group R vs. HCs), “cell cycle, “ “ubiquitin-mediated proteolysis, “ and “p53 signaling pathway” were significantly enriched among the candidate DEGs (**Figure 5F**).

**Figure 6:**
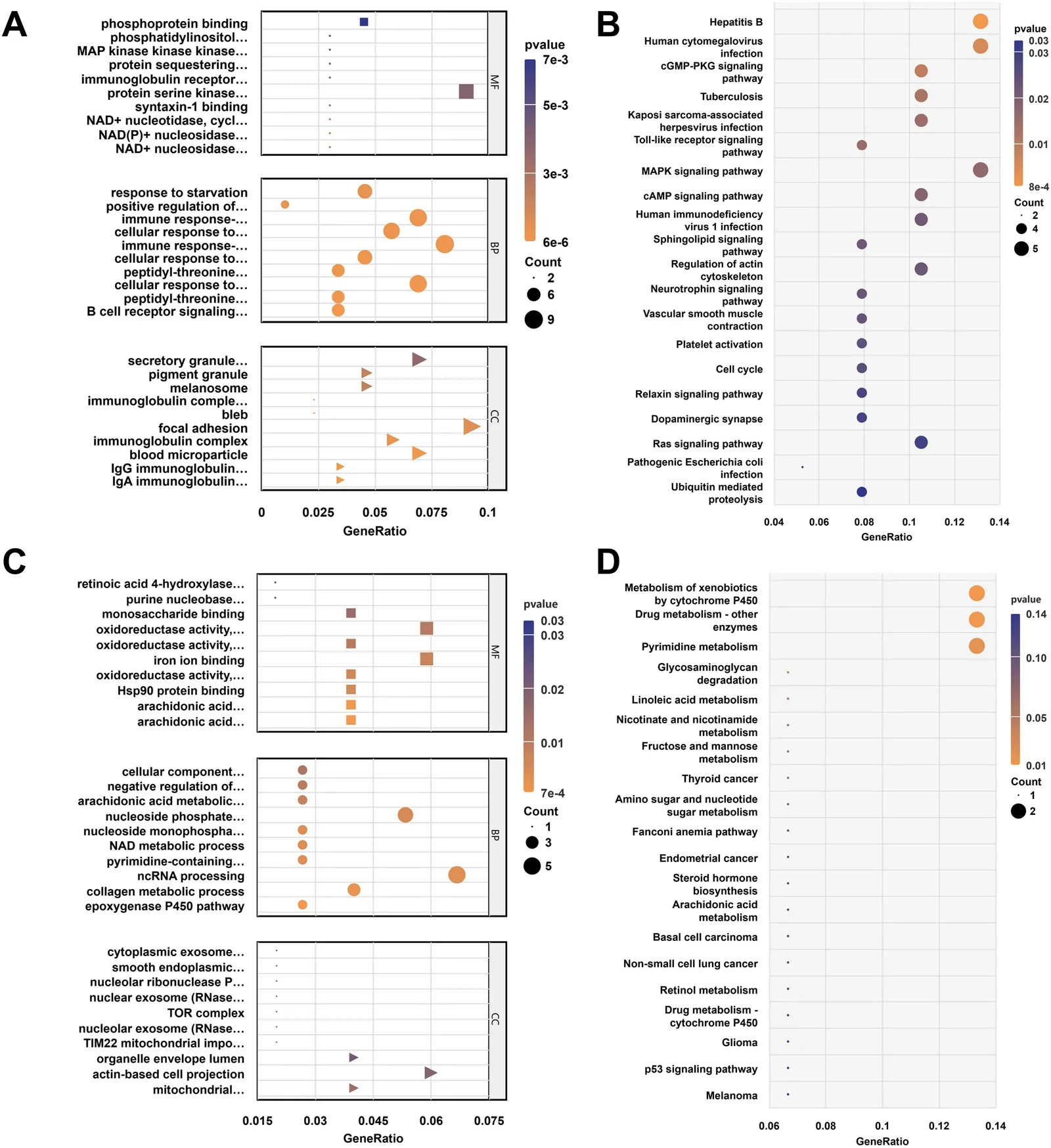
Functional enrichment analysis of candidate DEGs showing opposite stage-associated expression patterns. Samples included A group (n = 9), R group (n = 5), and HC group (n = 7). **(A, B)** GO and KEGG pathway enrichment analyses of 89 candidate DEGs showing higher expression in group A and lower expression in group R relative to HCs. **(C, D)** GO and KEGG pathway enrichment analyses of 68 candidate DEGs showing the opposite expression pattern. GO and KEGG enrichment was considered significant at *P* < 0.05.

Furthermore, 89 candidate DEGs showed higher expression in the acute-phase group than in HCs and lower expression in the remission-phase group than in HCs, whereas 68 candidate DEGs showed the opposite pattern. Subsequently, GO and KEGG enrichment analyses were performed to characterize the biological functions and pathways associated with these stage-associated expression patterns. For candidate DEGs showing higher expression in group A and lower expression in group R relative to HCs. GO MF analysis revealed that “protein serine kinase activity, “ “phosphoprotein binding, “ and “immunoglobulin receptor binding” were among the significantly enriched terms. GO BP analysis showed that “B cell receptor signaling pathway, “ “cellular response to external stimulus, “ and “immune response–activating cell surface receptor signaling pathway” (**Figure 6A**) were among the enriched processes. KEGG pathway enrichment analysis demonstrated that the candidate DEGs were significantly enriched in “toll-like receptor signaling pathway, “ “MAPK signaling pathway, “ and “sphingolipid signaling pathway” (**Figure 6B**). In the case of candidate DEGs upregulated in the remission phase and downregulated in the acute phase, GO MF analysis revealed that “arachidonic acid epoxygenase activity, “ “Hsp90 protein binding, “ and “oxidoreductase activity” were the significantly enriched terms. GO BP analysis showed that “epoxygenase P450 pathway, “ “collagen metabolic process, “ and “negative regulation of defense response” were among the enriched processes (**Figure 6C**). KEGG pathway enrichment analysis further indicated that these candidate DEGs were significantly enriched in pathways such as “metabolism of xenobiotics by cytochrome P450, “ “drug metabolism - other enzymes, “ and “pyrimidine metabolism” (**Figure 6D**). Complete GO and KEGG enrichment results for all comparisons are provided in **Supplementary Table 3**.

**Figure 7:**
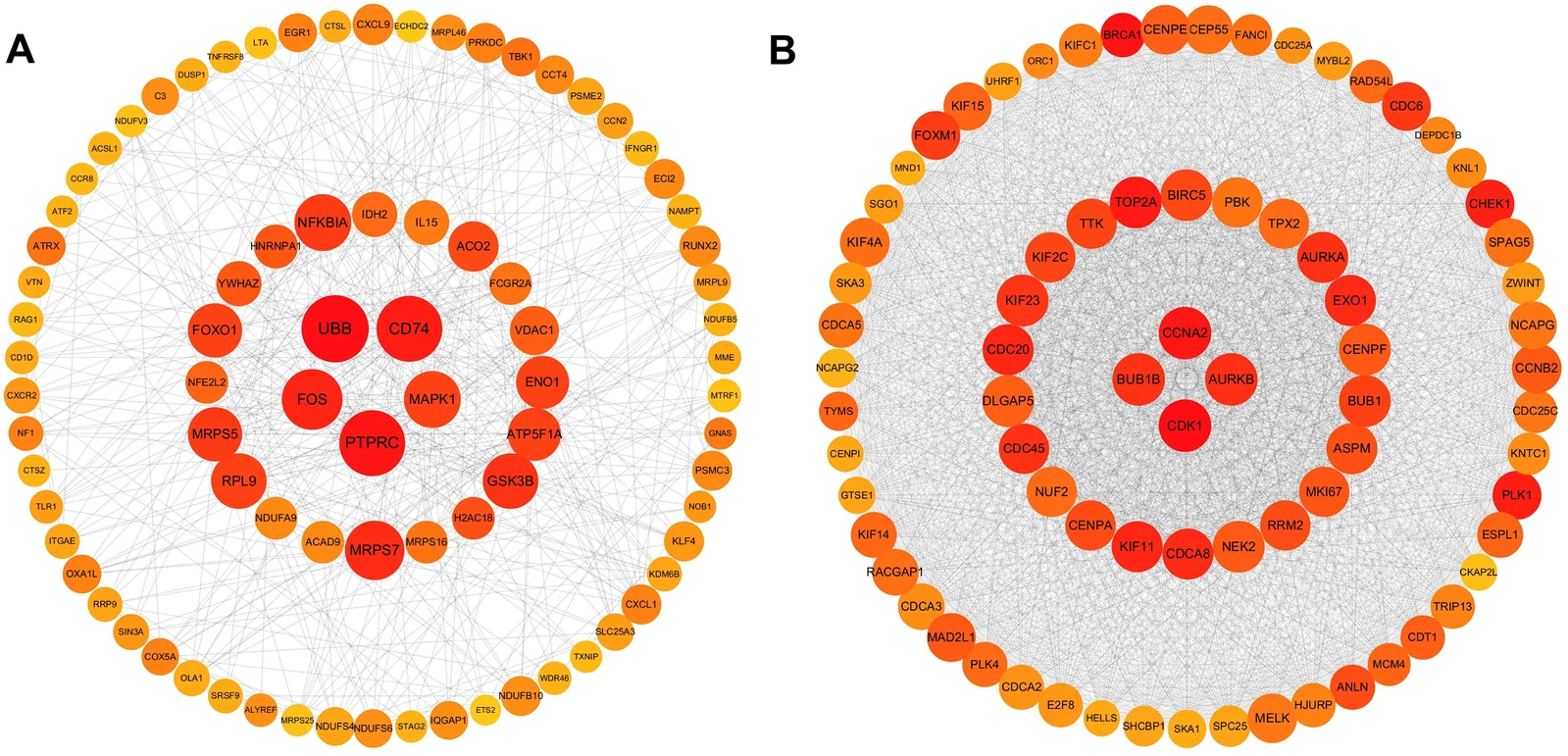
PPI network analysis of candidate DEGs. Samples included A group (n = 9), R group (n = 5), and HC group (n = 7). **(A)** PPI network analysis of candidate DEGs in group A and HC group. **(B)** PPI network analysis of candidate DEGs in group R and the HC group. Candidate hub genes were identified based on degree connectivity.

Finally, PPI network analysis of the candidate DEGs across the three groups was conducted using STRING and Cytoscape (**Figure 7**). The results identified *CD74* and *UBB* as candidate hub genes in the acute phase and *CDK1* as a candidate hub gene in the remission phase. These findings suggest that *CD74* and *CDK1* may be associated with stage-associated molecular features of MOG-ON; however, their biological and clinical relevance requires further validation.

### 3.6 Cell-type enrichment analysis based on gene expression signature

**Figure 8:**
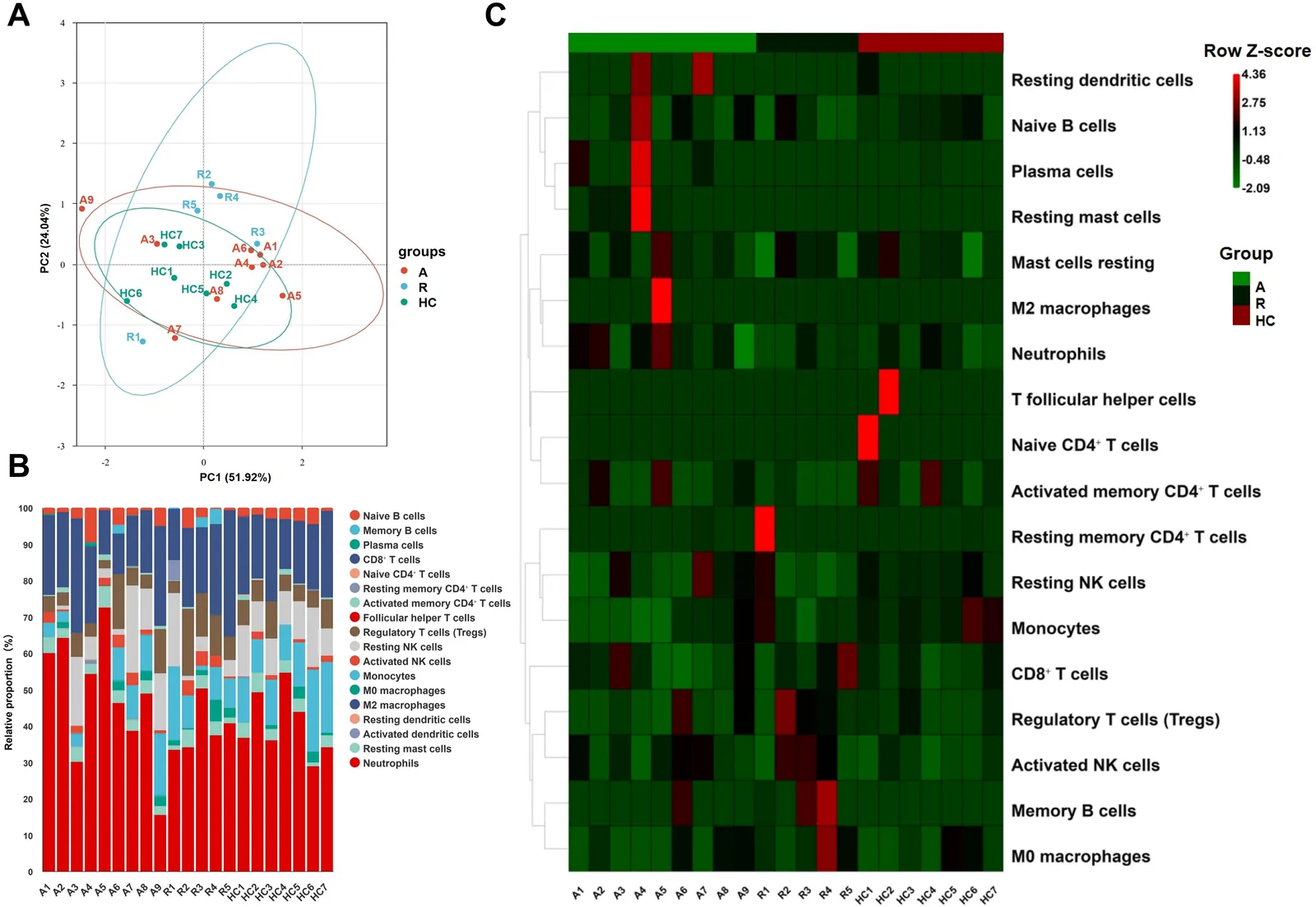
Analysis of estimated peripheral immune-cell proportions. Samples included A group (n = 9), R group (n = 5), and HC group (n = 7). **(A)** PCA analysis of estimated immune-cell composition across the three groups. **(B)** Stacked bar plot showing the relative proportions of immune-cell subsets in individual samples. **(C)** Heatmap showing the estimated proportions of immune-cell subsets in individual samples. Values are shown as row Z-scores. Immune-cell proportions were estimated using CIBERSORTx with the LM22 signature matrix.

The relative proportions of 22 peripheral immune-cell subsets in the three groups were estimated using CIBERSORTx (**Supplementary Table 4**). After removing four immune cell subsets with estimated proportions of zero (γδ T cells, M1 macrophages, activated mast cells, and eosinophils), the proportions of the remaining 18 immune-cell subsets in each group were evaluated. PCA revealed that the peripheral immune-cell composition profiles differed across the three groups (**Figure 8A**). Neutrophils and CD8^+^ T cells were the two most abundant immune cell populations. In the acute-phase group, the proportion of neutrophils was high in most samples, except A3 and A9. Although plasma cells, activated dendritic cells, and resting dendritic cells were detected only in acute-phase samples, activated CD4^+^ T cells were barely observed in remission samples. Moreover, memory B cells were present only in the disease groups, not in the HC group. The estimated proportions of immune cells in the remission group were similar to those in the HC group (**Figures 8B, C**).

### 3.7 Correlation analysis between estimated immune cell proportions and visual acuity at the last follow-up

**Figure 9:**
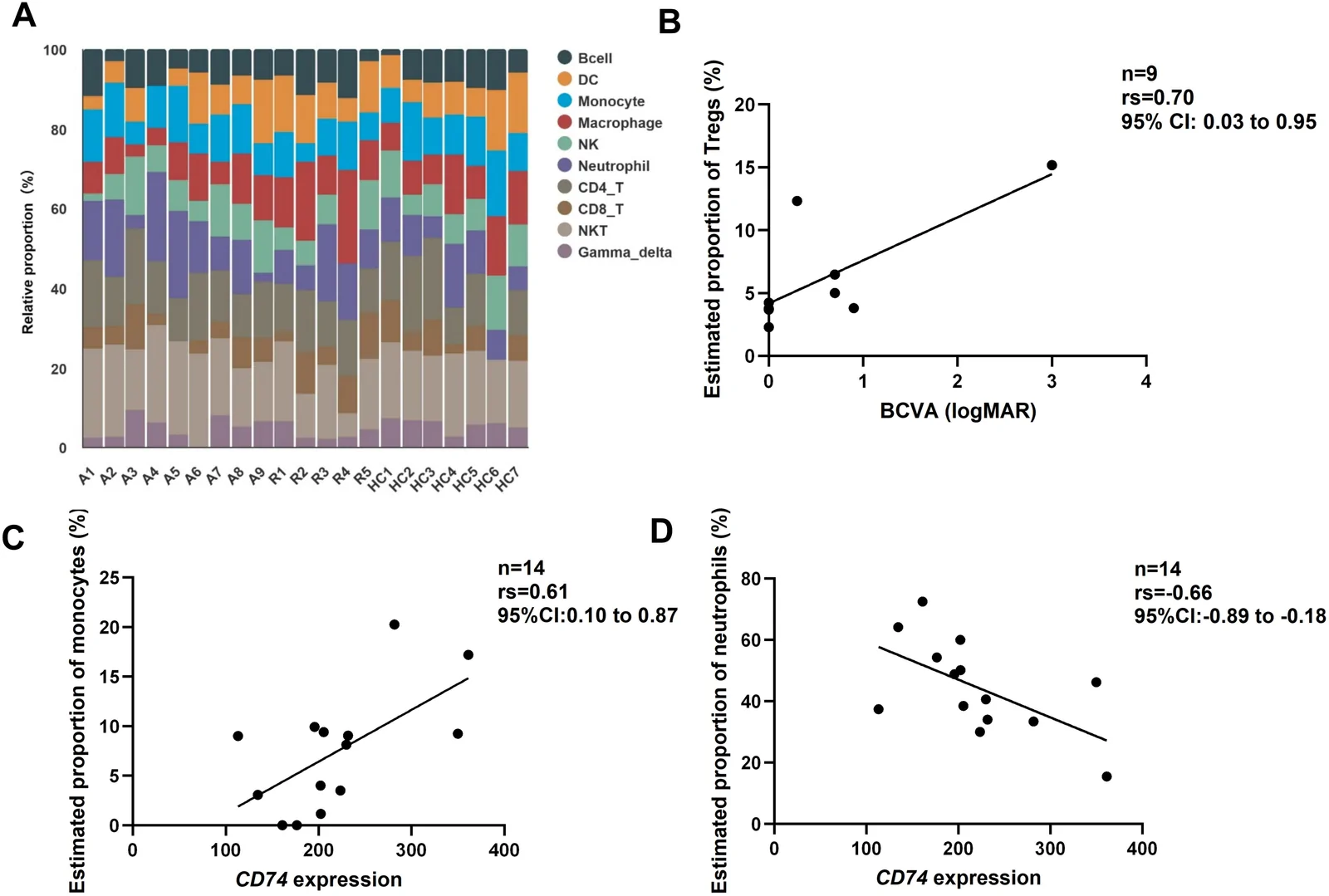
Estimated immune-cell composition and correlation analyses. **(A)** Immune-cell composition was estimated for all samples using ImmuCellAI. The stacked bar plot shows the relative proportions of 10 immune-cell categories in the MOG-ON and HC groups. The x-axis indicates individual sample IDs, and the y-axis indicates the relative proportion of each immune-cell category. Gamma_delta, γδ T cells; NKT, natural killer T cells; CD8_T, CD8^+^ T cells; CD4_T, CD4^+^ T cells; NK, natural killer cells; DC, dendritic cells. **(B)** Correlation between BCVA (logMAR) at the last follow-up and the estimated Treg proportion in group A (n = 9, *r*s = 0.70, 95% CI: 0.03 to 0.95, raw *P* = 0.04, q = 0.39). **(C)** Correlation between *CD74* expression and the estimated monocyte proportion (n = 14, *r*s = 0.61, 95% CI: 0.10 to 0.87, raw *P* = 0.02, q = 0.16). **(D)** Correlation between *CD74* expression and the estimated neutrophil proportion (n = 14, *r*s = −0.66, 95% CI: −0.89 to −0.18, raw *P* = 0.01, q = 0.16).

ImmuCellAI analysis showed broadly comparable patterns of estimated immune-cell composition across the MOG-ON and HC samples compared with those obtained using CIBERSORTx(**Figure 9A**). To further evaluate cross-method concordance, immune-cell estimates derived from CIBERSORTx and ImmuCellAI were compared for cell populations with comparable definitions across the two algorithms. Significant positive correlations were observed for neutrophils (*r_s_* = 0.89, *P* < 0.0001), CD8^+^ T cells (*r_s_* = 0.73, *P* = 0.0002), and NK cells (*r_s_* = 0.66, *P* = 0.001). In contrast, no significant cross-method correlation was observed for monocytes (*r_s_* = 0.01, *P* = 0.95) (**Supplementary Table 5**; **Supplementary Figure 1**).

The Spearman correlation analysis was performed between the estimated immune-cell proportions calculated using CIBERSORTx and the BCVA converted to logMAR at the last follow-up. In the acute-phase group, the proportion of regulatory T cells (Tregs) showed a positive association with BCVA (n=9, *r_s_* = 0.70, 95% CI: 0.03 to 0.95, raw *P* = 0.04); however, this association did not remain significant after Benjamini–Hochberg correction (*q* = 0.39) (**Figure 9B**). No significant correlations were identified in the remission-phase group. In addition, correlations between *CD74* gene expression and the estimated immune-cell proportions were examined. *CD74* expression was negatively correlated with neutrophils (n=14, *r_s_* =−0.66, 95% CI: -0.89 to -0.18, raw *P* = 0.01), and positively correlated with monocytes (n=14, *r_s_* = 0.61, 95% CI: 0.10 to 0.87, raw *P* = 0.02); however, neither association remained significant after BH correction (both *q* = 0.16) (**Figures 9C, D**). Complete results of the correlation analyses, including sample size, Spearman’s *r*s, 95% confidence intervals, raw *P* values, and BH-adjusted *P* values, are provided in **Supplementary Table 6**. Overall, the correlation analyses suggested potential associations between peripheral immune-cell composition, *CD74* expression, and visual outcomes.

## 4 Discussion

Previous studies of MOG-ON have primarily focused on immune mechanisms, including autoantibody production, complement activation, and inflammatory cell infiltration. However, its post-transcriptional regulation has not been adequately investigated (24–27). This study is the first to provide evidence of altered A-to-I RNA editing in MOG-ON, identify distinct immunoregulatory profiles across disease stages, and suggest a potential link between peripheral immune cell composition and visual outcomes. The findings offer novel insights into the pathogenesis of MOG-ON and may aid in identifying candidate biomarkers potentially associated with disease activity and visual outcomes, thereby providing a basis for future studies on patient stratification and individualized clinical monitoring.

A-to-I RNA editing has been implicated in maintaining immune tolerance to endogenous RNA and regulating T-cell development (28–30). In this study, differentially RNA-edited genes were significantly enriched in neutrophil-mediated immune responses and the regulation of T-cell differentiation. Therefore, altered RNA editing may be associated with both innate and adaptive immune responses in MOG-ON (31, 32). Interaction network analysis identified that *ITGAX* and *C5AR1* were the candidate hub genes within the differentially RNA-edited gene network. *ITGAX* encodes CD11c, which plays a major role in myeloid cell adhesion, migration, and antigen processing and presentation. These processes potentially link innate immune activation to T-cell responses (33–35). *C5AR1* encodes complement C5a receptor 1, which mediates neutrophil chemotaxis, activation, and amplification of inflammatory responses (36–38). Based on the functional enrichment and network analyses, RNA-editing alterations involving ITGAX and C5AR1 may be associated with myeloid cell activation, complement-related neutrophilic inflammation, and T-cell-related immune responses (39). However, these network-based associations are exploratory and require validation in larger independent cohorts and functional studies.

The immunoregulatory profiles of MOG-ON differed between the acute and remission phases. The acute phase was primarily enriched in B-cell receptor signaling and immunoglobulin-mediated immune responses, consistent with increased humoral immune activity (40, 41). PPI analysis identified that *CD74* was a candidate hub gene in the acute phase and that its expression was linked to the relative proportions of neutrophils and monocytes. As an MHC class II invariant chain, *CD74* participates in antigen processing and presentation and may be involved in interactions between myeloid antigen-presenting cells and CD4^+^ T cells (42–44). Moreover, cell-surface *CD74* functions as a component of the MIF receptor complex, stimulating inflammatory cell activation, migration, and cytokine release via ERK/MAPK and NF-κB signaling (45–47). These findings suggest that *CD74* may represent a candidate marker associated with acute immune activation in MOG-ON, although its therapeutic relevance requires further investigation. In contrast, the remission phase was significantly enriched in cell cycle–related pathways, and CDK1 was identified as a central candidate hub gene. As a key kinase regulating cell-cycle progression and proliferation, *CDK1* suggests a shift in molecular activity in MOG-ON from immune activation to cell proliferation, tissue homeostasis restoration, and post-injury remodeling as acute inflammation subsides (17). However, as most samples in the acute phase and remission phase were unpaired and the remission-phase patients had received immunosuppressive therapy, these findings should not be interpreted as within-patient transitions from immune activation to tissue repair. Longitudinal studies with functional validation are needed to clarify the biological significance of *CD74* and *CDK1* in MOG-ON.

Unlike the acute phase of AQP4-ON, which has been reported to show enrichment of Toll-like receptor, cytokine, and neutrophil-related pathways, acute MOG-ON in the present study showed enrichment of B-cell receptor and immunoglobulin-mediated immune responses, suggesting distinct immune-related pathway patterns between the two disorders (16, 17). Previous findings in AQP4-ON have highlighted innate immune and neutrophil-related inflammatory signals, whereas the present MOG-ON data showed more prominent B-cell- and humoral immunity-related features during the acute phase (40). *CDK1* was identified as a candidate hub gene during remission in both AQP4-ON and MOG-ON, suggesting that the two disorders may share cell-cycle regulatory features during this phase.

Analysis of estimated immune-cell proportions suggested potential associations between peripheral immune-cell composition and visual outcomes in MOG-ON. In the acute phase, the estimated proportion of Tregs showed a nominal positive association with BCVA at the last follow-up. Given the well-established immunoregulatory and protective functions of Tregs in CNS autoimmunity, this trend may reflect a compensatory regulatory response to a greater inflammatory burden rather than a direct detrimental effect of Tregs (32, 48, 49). *CD74* is highly relevant to the antigen-presenting function of monocytes. The nominal positive correlation between *CD74* expression and monocyte proportion may therefore reflect enhanced monocyte-associated antigen-presentation activity (50), whereas its inverse correlation with neutrophils may indicate a relative shift from neutrophil-dominant innate inflammatory responses toward monocyte-associated immune regulation and antigen presentation (51–53). These exploratory findings suggest that peripheral immune-cell composition may be related to visual outcomes in MOG-ON, warranting further validation in larger longitudinal cohorts (54).

Overall, whole-blood transcriptomic analysis helped characterize the RNA editome of MOG-ON and delineate stage-associated immune regulatory features and candidate genes during the acute and remission phases. Exploratory correlation analyses suggested potential relationships between estimated immune-cell proportions and visual outcomes. However, this study has a few limitations. First, the sample size was relatively small, primarily owing to the rarity of MOG-ON and the limited number of eligible patients available at a single center. In addition, loss to follow-up and difficulties in repeated post-treatment blood sampling restricted the collection of paired longitudinal samples. As only two patients provided paired samples across both disease stages, most acute–remission comparisons were cross-sectional and may have been influenced by interindividual variability, including differences in disease duration and prior treatment exposure. Therefore, the observed molecular differences should be interpreted as preliminary stage-associated group differences that may also reflect treatment-related effects, rather than within-patient temporal changes. Second, estimated immune-cell proportions were derived from peripheral blood collected at the study sampling time point, whereas BCVA was assessed at the last follow-up. This temporal separation precludes determination of the directionality of these associations and does not permit causal inference. Third, transcriptomic profiling was performed exclusively on peripheral blood samples, which are susceptible to systemic confounding factors and exhibit substantial interindividual heterogeneity. Without matched genomic DNA, rare germline variants cannot be completely excluded, and paired DNA–RNA sequencing or targeted Sanger sequencing is needed for validation. Finally, this work provides only correlational evidence and lacks functional data from *in vitro* cellular assays or *in vivo* animal studies. Accordingly, the present study should be considered preliminary and exploratory. The identified RNA-editing alterations and candidate genes require confirmation in larger independent cohorts using orthogonal experimental approaches.

## 5 Conclusion

In conclusion, this study identified distinct A-to-I RNA editing and immune-related profiles in MOG-ON. ITGAX and C5AR1 were identified as candidate RNA editing–related genes, while CD74 and CDK1 were associated with the acute and remission phases, respectively. Exploratory analyses suggested potential associations between estimated peripheral immune-cell proportions and visual outcomes. These findings require further validation in larger independent cohorts.

## Data Availability

The datasets presented in this study can be found in online repositories. The names of the repository/repositories and accession number(s) can be found in the article/**Supplementary Material**.

## References

[1] Banwell B Bennett JL Marignier R Kim HJ Brilot F Flanagan EP . Diagnosis of myelin oligodendrocyte glycoprotein antibody-associated disease: International MOGAD Panel proposed criteria. Lancet Neurol. (2023) 22:268–82. doi: 10.1016/s1474-4422(22)00431-8 36706773

[2] Häußler V Trebst C Engels D Pellkofer H Havla J Duchow A . Real-world multicentre cohort study on choices and effectiveness of immunotherapies in NMOSD and MOGAD. J Neurol Neurosurg Psychiatry. (2025) 96:582–92. doi: 10.1136/jnnp-2024-334764 39730197 PMC12171486

[3] Andersen J Brilot F . Myelin oligodendrocyte glycoprotein antibody-associated disease (MOGAD): Insights into pathogenesis and biomarkers of prognosis. Semin Immunol. (2025) 78:101944. doi: 10.1016/j.smim.2025.101944 40088708

[4] Trewin BP Brilot F Reddel SW Dale RC Ramanathan S . MOGAD: A comprehensive review of clinicoradiological features, therapy and outcomes in 4699 patients globally. Autoimmun Rev. (2025) 24:103693. doi: 10.1016/j.autrev.2024.103693 39577549

[5] Al-Ani A Chen JJ Costello F . Myelin oligodendrocyte glycoprotein antibody-associated disease (MOGAD): current understanding and challenges. J Neurol. (2023) 270:4132–50. doi: 10.1007/s00415-023-11737-8 37154894 PMC10165591

[6] Honghua Y . Advancing visual neuroscience: expanding boundaries from bench to bedside. Visual Neurosci. (2025) 42:vns-0024-0001. doi: 10.48130/vns-0024-0001

[7] Corbali O Chitnis T . Pathophysiology of myelin oligodendrocyte glycoprotein antibody disease. Front Neurol. (2023) 14:1137998. doi: 10.3389/fneur.2023.1137998 36925938 PMC10011114

[8] Huang Y Zhu S Yao S Zhai H Liu C Han JJ . Unraveling aging from transcriptomics. Trends Genet. (2025) 41:218–35. doi: 10.1016/j.tig.2024.09.006 39424502

[9] Martinez-Usatorre A Ciarloni L Angelino P Wosika V Conforte AJ Fonseca Costa SS . Human blood cell transcriptomics unveils dynamic systemic immune modulation along colorectal cancer progression. J Immunother Cancer. (2024) 12:e009888. doi: 10.1136/jitc-2024-009888 39577870 PMC11590809

[10] Kueckelhaus J Frerich S Kada-Benotmane J Koupourtidou C Ninkovic J Dichgans M . Inferring histology-associated gene expression gradients in spatial transcriptomic studies. Nat Commun. (2024) 15:7280. doi: 10.1038/s41467-024-50904-x 39179527 PMC11343836

[11] Cao Y Yao W Chen F . Brief research report: WGCNA-driven identification of histone modification genes as potential biomarkers in AQP4-Associated optic neuritis. Front Genet. (2024) 15:1423584. doi: 10.3389/fgene.2024.1423584 39238786 PMC11374599

[12] Liu P Chen W Jiang H Huang H Liu L Fang F . Differential effects of SARM1 inhibition in traumatic glaucoma and EAE optic neuropathies. Mol Ther Nucleic Acids. (2023) 32:13–27. doi: 10.1016/j.omtn.2023.02.029 36950280 PMC10025007

[13] Kaneko K Kuroda H Matsumoto Y Sakamoto N Yamazaki N Yamamoto N . Different complement activation patterns following C5 cleavage in MOGAD and AQP4-IgG+NMOSD. Neurol Neuroimmunol Neuroinflamm. (2024) 11:e200293. doi: 10.1212/nxi.0000000000200293 39133885 PMC11379436

[14] Kim HJ Lee EJ Kim SY Kim H Kim KW Kim S . Serum proteins for monitoring and predicting visual function in patients with recent optic neuritis. Sci Rep. (2023) 13:5609. doi: 10.1038/s41598-023-32748-5 37019946 PMC10076295

[15] Werry EL Bright FM Piguet O Ittner LM Halliday GM Hodges JR . Recent developments in TSPO PET imaging as a biomarker of neuroinflammation in neurodegenerative disorders. Int J Mol Sci. (2019) 20:3161. doi: 10.3390/ijms20133161 31261683 PMC6650818

[16] Chen X Cheng L Pan Y Chen P Luo Y Li S . Different immunological mechanisms between AQP4 antibody-positive and MOG antibody-positive optic neuritis based on RNA sequencing analysis of whole blood. Front Immunol. (2023) 14:1095966. doi: 10.3389/fimmu.2023.1095966 36969199 PMC10036921

[17] Shi R Luo Y Chen Y Chen X Li S Chen Z . The different immune response between onset and remission of AQP4 antibody-positive optic neuritis based on RNA sequencing of whole blood. J Inflammation Res. (2025) 18:10283–93. doi: 10.2147/jir.S507083 40756415 PMC12318522

[18] Koboldt DC Zhang Q Larson DE Shen D Mclellan MD Lin L . VarScan 2: somatic mutation and copy number alteration discovery in cancer by exome sequencing. Genome Res. (2012) 22:568–76. doi: 10.1101/gr.129684.111 22300766 PMC3290792

[19] Tao J Ren CY Wei ZY Zhang F Xu J Chen JH . Transcriptome-wide identification of G-to-A RNA editing in chronic social defeat stress mouse models. Front Genet. (2021) 12:680548. doi: 10.3389/fgene.2021.680548 34093668 PMC8173075

[20] Jin YY Guo Y Xiong SW Zhang N Chen JH Liu F . BALF editome profiling reveals A-to-I RNA editing associated with severity and complications of Mycoplasma pneumoniae pneumonia in children. mSphere. (2025) 10:e0101224. doi: 10.1128/msphere.01012-24 39998235 PMC11934315

[21] Mansi L Tangaro MA Lo Giudice C Flati T Kopel E Schaffer AA . REDIportal: millions of novel A-to-I RNA editing events from thousands of RNAseq experiments. Nucleic Acids Res. (2021) 49:D1012–d1019. doi: 10.1093/nar/gkaa916 33104797 PMC7778987

[22] Kong FS Feng J Yao JP Lu Y Guo T Sun M . Dysregulated RNA editing of EIF2AK2 in polycystic ovary syndrome: clinical relevance and functional implications. BMC Med. (2024) 22:229. doi: 10.1186/s12916-024-03434-8 38853264 PMC11163819

[23] Nishikura K . Functions and regulation of RNA editing by ADAR deaminases. Annu Rev Biochem. (2010) 79:321–49. doi: 10.1146/annurev-biochem-060208-105251 20192758 PMC2953425

[24] Sechi E Cacciaguerra L Chen JJ Mariotto S Fadda G Dinoto A . Myelin oligodendrocyte glycoprotein antibody-associated disease (MOGAD): A review of clinical and MRI features, diagnosis, and management. Front Neurol. (2022) 13:885218. doi: 10.3389/fneur.2022.885218 35785363 PMC9247462

[25] Satukijchai C Mariano R Messina S Sa M Woodhall MR Robertson NP . Factors associated with relapse and treatment of myelin oligodendrocyte glycoprotein antibody-associated disease in the United Kingdom. JAMA Netw Open. (2022) 5:e2142780. doi: 10.1001/jamanetworkopen.2021.42780 35006246 PMC8749481

[26] Chen JJ Huda S Hacohen Y Levy M Lotan I Wilf-Yarkoni A . Association of maintenance intravenous immunoglobulin with prevention of relapse in adult myelin oligodendrocyte glycoprotein antibody-associated disease. JAMA Neurol. (2022) 79:518–25. doi: 10.1001/jamaneurol.2022.0489 35377395 PMC8981066

[27] Marignier R Hacohen Y Cobo-Calvo A Pröbstel AK Aktas O Alexopoulos H . Myelin-oligodendrocyte glycoprotein antibody-associated disease. Lancet Neurol. (2021) 20:762–72. doi: 10.1016/s1474-4422(21)00218-0 34418402

[28] Liddicoat BJ Piskol R Chalk AM Ramaswami G Higuchi M Hartner JC . RNA editing by ADAR1 prevents MDA5 sensing of endogenous dsRNA as nonself. Science. (2015) 349:1115–20. doi: 10.1126/science.aac7049 26275108 PMC5444807

[29] Sun T Li Q Geisinger JM Hu SB Fan B Su S . ADAR1 editing is necessary for only a small subset of cytosolic dsRNAs to evade MDA5-mediated autoimmunity. Nat Genet. (2025) 57:3101–11. doi: 10.1038/s41588-025-02430-9 41339703 PMC12695637

[30] Min DJ Lee S Lee YS Cho J . Two codes of RNA editing by deamination in human diseases. Exp Mol Med. (2026) 58:382–95. doi: 10.1038/s12276-025-01633-8 41708996 PMC12993061

[31] Baek SI Ro S Chung YH Ju H Kwon S Park KA . Novel index, neutrophil percentage (%) is a useful marker for disease activity in MOG antibody-associated disease. Mult Scler Relat Disord. (2023) 76:104796. doi: 10.1016/j.msard.2023.104796 37320937

[32] Horellou P De Chalus A Giorgi L Leroy C Chrétien P Hacein-Bey-Abina S . Regulatory T cells increase after rh-MOG stimulation in non-relapsing but decrease in relapsing MOG antibody-associated disease at onset in children. Front Immunol. (2021) 12:2021. doi: 10.3389/fimmu.2021.679770 34220827 PMC8243969

[33] Sándor N Lukácsi S Ungai-Salánki R Orgován N Szabó B Horváth R . CD11c/CD18 dominates adhesion of human monocytes, macrophages and dendritic cells over CD11b/CD18. PloS One. (2016) 11:e0163120. doi: 10.1371/journal.pone.0163120 27658051 PMC5033469

[34] Shirahama S Okunuki Y Lee MY Karg MM Refaian N Krasniqi D . Preventing the antigen-presenting function of retinal microglia blocks autoimmune neuroinflammation by dendritic cell-primed CD4(+) T cells. J Autoimmun. (2025) 153:103417. doi: 10.1016/j.jaut.2025.103417 40239533

[35] Shen X Qiu Y Wight AE Kim HJ Cantor H . Definition of a mouse microglial subset that regulates neuronal development and proinflammatory responses in the brain. Proc Natl Acad Sci USA. (2022) 119:e2116241119. doi: 10.1073/pnas.2116241119 35177477 PMC8872761

[36] Zhou J Ma S Feng D Tian Y Li L Guo H . C5aR1(+) microglia exacerbate neuroinflammation and cerebral edema in acute brain injury. Neuron. (2026) 114:444–462.e449. doi: 10.1016/j.neuron.2025.10.022 41260217

[37] Qi F Lennon VA Zhao S Guo Y Ding H Liu C . Neutrophil-microglia interaction drives motor dysfunction in a neuromyelitis optica model induced by subarachnoid AQP4-IgG. J Clin Invest. (2026) 136:e199706. doi: 10.1172/jci199706 41665955 PMC13038209

[38] Cristiano C Giorgio C Cocchiaro P Boccella S Cesta MC Castelli V . Inhibition of C5aR1 as a promising approach to treat taxane-induced neuropathy. Cytokine. (2023) 171:156370. doi: 10.1016/j.cyto.2023.156370 37722320

[39] Manouchehri N Hussain RZ Cravens PD Esaulova E Artyomov MN Edelson BT . CD11c(+)CD88(+)CD317(+) myeloid cells are critical mediators of persistent CNS autoimmunity. Proc Natl Acad Sci USA. (2021) 118:e201449211. doi: 10.1073/pnas.2014492118 33785592 PMC8040603

[40] Yan Y . Autoimmune optic neuropathy: Pathogenesis, diagnosis, and therapeutic advances. Int Ophthalmol Clin. (2026) 66:30–6. doi: 10.1097/iio.0000000000000596 41429673

[41] Tanaka K Kezuka T Ishikawa H Tanaka M Sakimura K Abe M . Pathogenesis, clinical features, and treatment of patients with myelin oligodendrocyte glycoprotein (MOG) autoantibody-associated disorders focusing on optic neuritis with consideration of autoantibody-binding sites: A review. Int J Mol Sci. (2023) 24:13368. doi: 10.3390/ijms241713368 37686172 PMC10488293

[42] Su H Na N Zhang X Zhao Y . The biological function and significance of CD74 in immune diseases. Inflammation Res. (2017) 66:209–16. doi: 10.1007/s00011-016-0995-1 27752708

[43] Li QL Tang J Zhao L Ruze A Shan XF Gao XM . The role of CD74 in cardiovascular disease. Front Cardiovasc Med. (2022) 9:1049143. doi: 10.3389/fcvm.2022.1049143 36712241 PMC9877307

[44] Wang D Zhang T Shao Q Wu X Zhao X Zhang H . GSDME-mediated pyroptosis in microglia exacerbates demyelination and neuroinflammation in multiple sclerosis: insights from humans and cuprizone-induced demyelination model mice. Cell Death Differ. (2025) 32:2368–83. doi: 10.1038/s41418-025-01537-0 40555745 PMC12669246

[45] Guan D Li Y Cui Y Zhao H Dong N Wang K . 5-HMF attenuates inflammation and demyelination in experimental autoimmune encephalomyelitis mice by inhibiting the MIF-CD74 interaction. Acta Biochim Biophys Sin (Shanghai). (2023) 55:1222–33. doi: 10.3724/abbs.2023105 37431183 PMC10448060

[46] Potru PS Spittau B . CD74: a prospective marker for reactive microglia? Neural Regener Res. (2023) 18:2673–4. doi: 10.4103/1673-5374.371350 37449617 PMC10358643

[47] Cao C Liu T Peng L Li L Xu Z Li X . Targeting CD74 in microglia to modulate experimental cerebral ischemia and reperfusion injury: insights from single-cell and bulk transcriptomics. Mol Brain. (2025) 18:46. doi: 10.1186/s13041-025-01197-8 40400029 PMC12096678

[48] Correale J Carnero Contentti E . Induction of immune tolerance in NMOSD and MOGAD. Ther Adv Neurol Disord. (2025) 18:17562864251357393. doi: 10.1177/17562864251357393 40761287 PMC12319201

[49] Ma X Qin C Chen M Yu HH Chu YH Chen TJ . Regulatory T cells protect against brain damage by alleviating inflammatory response in neuromyelitis optica spectrum disorder. J Neuroinflamm. (2021) 18:201. doi: 10.1186/s12974-021-02266-0 34526069 PMC8444427

[50] Monaghan KL Zheng W Hu G Wan ECK . Monocytes and monocyte-derived antigen-presenting cells have distinct gene signatures in experimental model of multiple sclerosis. Front Immunol. (2019) 10:2779. doi: 10.3389/fimmu.2019.02779 31849962 PMC6889845

[51] Liu J Yang X Pan J Wei Z Liu P Chen M . Single-cell transcriptome profiling unravels distinct peripheral blood immune cell signatures of RRMS and MOG antibody-associated disease. Front Neurol. (2021) 12:807646. doi: 10.3389/fneur.2021.807646 35095746 PMC8795627

[52] Wang L Xia R Li X Shan J Wang S . Systemic inflammation response index is a useful indicator in distinguishing MOGAD from AQP4-IgG-positive NMOSD. Front Immunol. (2023) 14:1293100. doi: 10.3389/fimmu.2023.1293100 38259484 PMC10800877

[53] Cheng X Sun Y Wang Y Cheng W Zhang H Jiang Y . The percentage of neutrophils is independently associated with blood-brain barrier(BBB) disruption in myelin oligodendrocyte glycoprotein antibody associated disease (MOGAD). J Inflammation Res. (2025) 18:2823–36. doi: 10.2147/jir.S501150 40026312 PMC11871905

[54] Shao Y Lou Y Gao Y Tan G Liu T Yu H . Expert consensus on the application of artificial intelligence in neuro-ophthalmic diseases. Visual Neurosci. (2025) 42:e019.

