# Correction: A-to-I RNA editing profiles and distinct immune responses in myelin oligodendrocyte glycoprotein antibody-positive optic neuritis

**DOI:** 10.3389/fimmu.2026.2004755

**Published:** 2026-10-06

**Authors:** Xueqing Cui, Jia-Qi Pan, Jianan Ding, Xuchu Fu, Fanli Cheng, Yutong Zhang, Jian-Huan Chen, Wenjun Zou

**Affiliations:** 1 Department of Ophthalmology, Jiangnan University Medical Center (JUMC), Wuxi, Jiangsu, China; 2 Department of Ophthalmology, Wuxi No. 2 People’s Hospital, Wuxi, Jiangsu, China; 3 Laboratory of Genomic and Precision Medicine, Wuxi School of Medicine, Jiangnan University, Wuxi, Jiangsu, China

**Keywords:** A-to-I RNA editing, gene expression, immune response, inflammation, MOG antibody-positive optic neuritis

Author Jia-Qi Pan and Jian-Huan Chen were omitted as authors in the published article. The correct author list reads:

“Xueqing Cui^1,2^, Jia-Qi Pan^3^, Jianan Ding^1,2^, Xuchu Fu^1,2^, Fanli Cheng^1,2^, Yutong Zhang^1,2^, Jian-Huan Chen^3^ and Wenjun Zou^1,2*^”

“1 Department of Ophthalmology, Jiangnan University Medical Center (JUMC), Wuxi, Jiangsu, China; 2 Department of Ophthalmology, Wuxi No. 2 People’s Hospital, Wuxi, Jiangsu, China; 3 Laboratory of Genomic and Precision Medicine, Wuxi School of Medicine, Jiangnan University, Wuxi, Jiangsu, China”

The **Author contributions** Statement has been corrected to read:

“XC: Writing – review & editing, Visualization, Data curation, Formal analysis, Methodology, Writing – original draft, Investigation, Conceptualization. JP: Writing – original draft, Formal analysis, Software, Data curation, Visualization. JD: Visualization, Formal analysis, Investigation, Writing – original draft. XF: Writing – original draft, Formal analysis, Investigation. FC: Formal analysis, Visualization, Writing – original draft. YZ: Formal analysis, Writing – original draft, Visualization. JC: Writing – review & editing, Data curation, Methodology, Supervision, Conceptualization, Formal analysis. WZ: Methodology, Supervision, Project administration, Funding acquisition, Writing – review & editing, Conceptualization.”

The original version of this article has been updated.

