# Supplementary material for: A-to-I RNA editing profiles and distinct immune responses in myelin oligodendrocyte glycoprotein antibody-positive optic neuritis
Source: Front Immunol. 2026 Sep 3;17:1946792. doi: 10.3389/fimmu.2026.1946792 (PMC13582522; doi:10.3389/fimmu.2026.1946792)
Supplement: Supplementary file 1 [file DataSheet1.pdf]

SUPPLEMENTARY FIGURE 1 Cross-method concordance of immune-cell estimates derived from CIBERSORTx and ImmuCellAI.

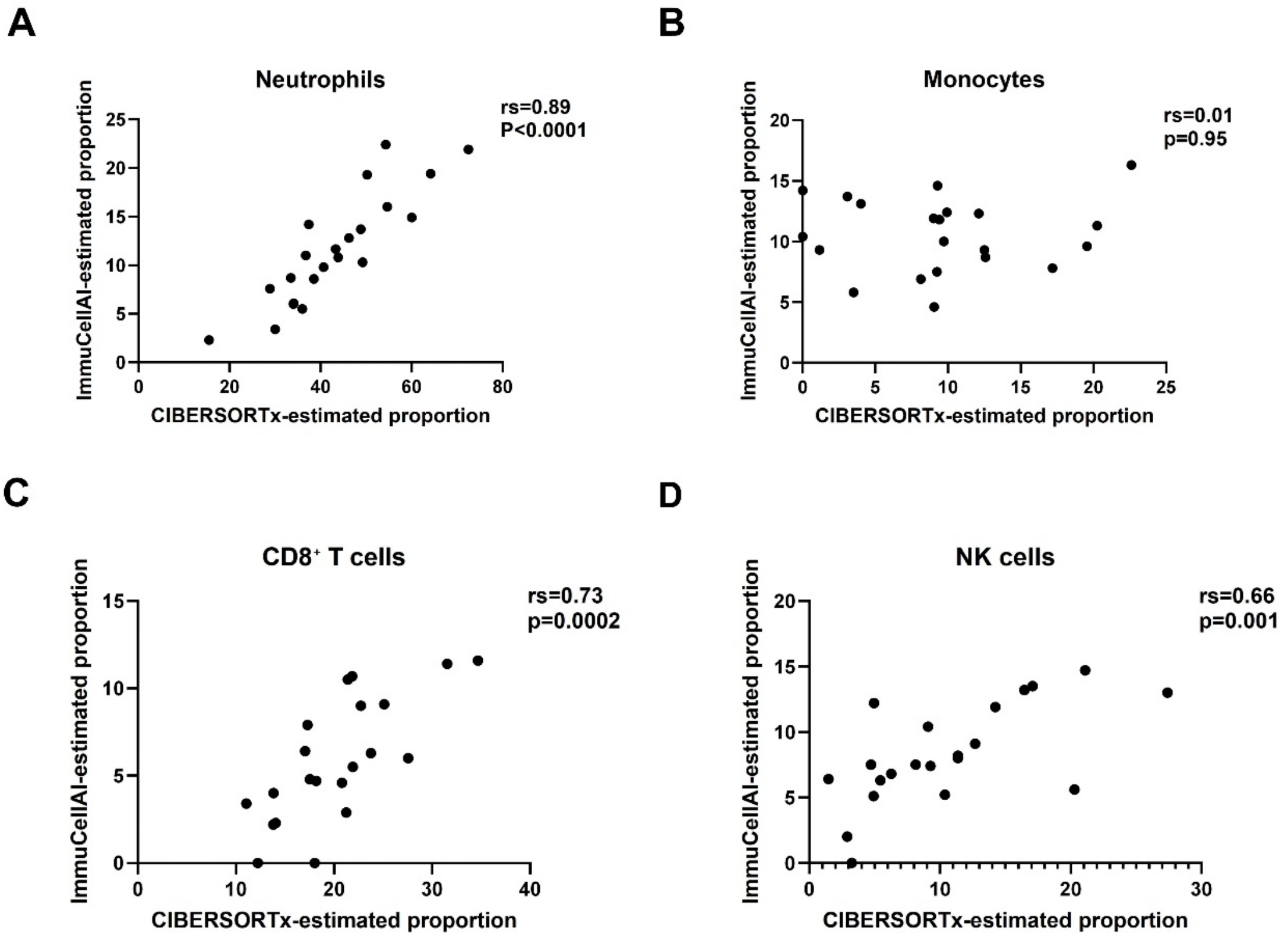

Scatter plots show the correlations between immune-cell proportions estimated by CIBERSORTx and ImmuCellAI across 21 matched samples for (A) neutrophils, (B) monocytes, (C) $CD8^+$ T cells, and (D) NK cells. Spearman correlation analysis was performed for each cell type. For NK cells, the resting and activated NK-cell fractions estimated by CIBERSORTx were summed before comparison with the NK-cell estimates generated by ImmuCellAI.
