# Supplementary material for: A-to-I RNA editing profiles and distinct immune responses in myelin oligodendrocyte glycoprotein antibody-positive optic neuritis
Source: Front Immunol. 2026 Sep 3;17:1946792. doi: 10.3389/fimmu.2026.1946792 (PMC13582522; doi:10.3389/fimmu.2026.1946792)
Supplement: Supplementary file 2 [file Table4.docx]

**Supplement Table 4.** Estimated immune cell proportions and CIBERSORTx deconvolution quality metrics for each sample

| **Cell type** | **HC1** | **HC2** | **HC3** | **HC4** | **HC5** | **HC6** | **HC7** | **A1** | **A2** | **A3** | **A4** | **A5** | **A6** | **A7** | **A8** | **A9** | **R1** | **R2** | **R3** | **R4** | **R5** |
| --- | --- | --- | --- | --- | --- | --- | --- | --- | --- | --- | --- | --- | --- | --- | --- | --- | --- | --- | --- | --- | --- |
| Naive B cells | 2.37% | 1.93% | 2.92% | 3.07% | 3.73% | 4.48% | 0.95% | 1.71% | 1.19% | 2.97% | 9.60% | 0.63% | 4.68% | 2.05% | 0.68% | 5.00% | 0.00% | 5.54% | 2.53% | 0.30% | 0.74% |
| Memory B cells | 0.00% | 0.00% | 0.00% | 0.00% | 0.00% | 0.00% | 0.00% | 0.00% | 0.00% | 0.00% | 0.00% | 0.00% | 2.53% | 0.00% | 0.00% | 0.00% | 0.40% | 0.00% | 2.84% | 4.16% | 0.00% |
| Plasma cells | 0.00% | 0.00% | 0.00% | 0.04% | 0.00% | 0.00% | 0.00% | 0.42% | 0.00% | 0.00% | 0.94% | 0.00% | 0.00% | 0.18% | 0.00% | 0.00% | 0.00% | 0.00% | 0.00% | 0.00% | 0.00% |
| CD8⁺ T cells | 21.42% | 17.54% | 22.75% | 13.80% | 17.05% | 18.03% | 23.76% | 21.93% | 20.81% | 31.55% | 21.25% | 12.23% | 11.06% | 13.83% | 17.31% | 27.65% | 14.06% | 21.85% | 18.18% | 25.12% | 34.68% |
| Naive CD4⁺ T cells | 0.18% | 0.00% | 0.00% | 0.00% | 0.00% | 0.00% | 0.00% | 0.00% | 0.00% | 0.00% | 0.00% | 0.00% | 0.00% | 0.00% | 0.00% | 0.00% | 0.00% | 0.00% | 0.00% | 0.00% | 0.00% |
| Resting memory CD4⁺ T cells | 0.00% | 0.00% | 0.00% | 0.00% | 0.00% | 0.19% | 0.00% | 0.00% | 0.00% | 0.00% | 0.00% | 0.00% | 0.00% | 0.31% | 0.00% | 0.00% | 5.53% | 0.00% | 0.00% | 0.00% | 0.00% |
| Activated memory CD4⁺ T cells | 1.48% | 0.47% | 0.00% | 1.57% | 0.49% | 0.00% | 0.47% | 0.36% | 1.24% | 0.00% | 0.00% | 1.50% | 0.00% | 0.06% | 0.42% | 0.76% | 0.12% | 0.38% | 0.00% | 0.00% | 0.00% |
| Follicular helper T cells | 0.00% | 0.18% | 0.00% | 0.00% | 0.00% | 0.00% | 0.00% | 0.00% | 0.00% | 0.00% | 0.00% | 0.00% | 0.00% | 0.00% | 0.00% | 0.00% | 0.00% | 0.00% | 0.00% | 0.00% | 0.00% |
| Regulatory T cells (Tregs) | 6.86% | 5.63% | 10.35% | 4.40% | 4.42% | 4.78% | 8.10% | 4.24% | 3.77% | 6.47% | 3.69% | 2.29% | 15.17% | 5.00% | 3.81% | 12.33% | 3.33% | 18.41% | 11.86% | 11.05% | 6.56% |
| Gamma delta T cells | 0.00% | 0.00% | 0.00% | 0.00% | 0.00% | 0.00% | 0.00% | 0.00% | 0.00% | 0.00% | 0.00% | 0.00% | 0.00% | 0.00% | 0.00% | 0.00% | 0.00% | 0.00% | 0.00% | 0.00% | 0.00% |
| Resting NK cells | 14.03% | 8.33% | 10.08% | 9.28% | 10.55% | 16.32% | 7.48% | 0.08% | 0.98% | 19.00% | 6.27% | 2.52% | 1.55% | 23.94% | 11.30% | 15.50% | 20.29% | 1.29% | 4.02% | 0.00% | 4.42% |
| Activated NK cells | 0.20% | 2.06% | 1.28% | 0.00% | 0.84% | 0.77% | 1.61% | 2.84% | 0.49% | 2.10% | 0.00% | 2.21% | 3.39% | 3.47% | 1.39% | 0.96% | 0.00% | 4.16% | 4.11% | 3.26% | 0.53% |
| Monocytes | 12.58% | 9.28% | 12.50% | 9.72% | 12.12% | 22.62% | 19.56% | 4.00% | 3.07% | 3.50% | 0.00% | 0.00% | 9.24% | 9.41% | 9.92% | 17.19% | 20.26% | 9.05% | 1.16% | 9.01% | 8.13% |
| M0 macrophages | 0.00% | 0.00% | 1.04% | 0.00% | 3.34% | 2.92% | 0.72% | 0.00% | 1.54% | 0.00% | 0.00% | 0.00% | 2.64% | 0.00% | 2.68% | 2.77% | 1.46% | 0.31% | 1.42% | 5.86% | 2.73% |
| M1 macrophages | 0.00% | 0.00% | 0.00% | 0.00% | 0.00% | 0.00% | 0.00% | 0.00% | 0.00% | 0.00% | 0.00% | 0.00% | 0.00% | 0.00% | 0.00% | 0.00% | 0.00% | 0.00% | 0.00% | 0.00% | 0.00% |
| M2 macrophages | 0.00% | 0.00% | 0.00% | 0.00% | 0.00% | 0.00% | 0.00% | 0.00% | 0.00% | 0.00% | 0.00% | 0.11% | 0.00% | 0.00% | 0.00% | 0.00% | 0.00% | 0.00% | 0.00% | 0.00% | 0.00% |
| Resting dendritic cells | 0.06% | 0.00% | 0.00% | 0.00% | 0.00% | 0.00% | 0.00% | 0.00% | 0.00% | 0.00% | 0.17% | 0.01% | 0.00% | 0.19% | 0.00% | 0.00% | 0.00% | 0.00% | 0.00% | 0.00% | 0.00% |
| Activated dendritic cells | 0.00% | 0.00% | 0.00% | 0.00% | 0.00% | 0.00% | 0.00% | 0.00% | 0.16% | 0.16% | 1.07% | 0.05% | 0.00% | 0.00% | 0.00% | 0.00% | 0.00% | 0.00% | 0.00% | 0.00% | 0.00% |
| Resting mast cells | 4.08% | 5.38% | 3.11% | 3.48% | 3.62% | 1.02% | 3.30% | 4.40% | 2.58% | 4.23% | 2.72% | 5.91% | 3.50% | 3.02% | 3.69% | 2.45% | 1.10% | 5.00% | 3.69% | 3.83% | 1.57% |
| Activated mast cells | 0.00% | 0.00% | 0.00% | 0.00% | 0.00% | 0.00% | 0.00% | 0.00% | 0.00% | 0.00% | 0.00% | 0.00% | 0.00% | 0.00% | 0.00% | 0.00% | 0.00% | 0.00% | 0.00% | 0.00% | 0.00% |
| Eosinophils | 0.00% | 0.00% | 0.00% | 0.00% | 0.00% | 0.00% | 0.00% | 0.00% | 0.00% | 0.00% | 0.00% | 0.00% | 0.00% | 0.00% | 0.00% | 0.00% | 0.00% | 0.00% | 0.00% | 0.00% | 0.00% |
| Neutrophils | 36.72% | 49.21% | 35.96% | 54.64% | 43.83% | 28.87% | 34.05% | 60.02% | 64.15% | 30.03% | 54.29% | 72.47% | 46.23% | 38.52% | 48.81% | 15.48% | 33.46% | 34.02% | 50.18% | 37.42% | 40.64% |
| P-value | <0.001 | <0.001 | <0.001 | <0.001 | <0.001 | <0.001 | <0.001 | <0.001 | <0.001 | <0.001 | <0.001 | <0.001 | <0.001 | <0.001 | <0.001 | <0.001 | <0.001 | <0.001 | <0.001 | <0.001 | <0.001 |
| Correlation | 0.82 | 0.85 | 0.64 | 0.72 | 0.87 | 0.64 | 0.67 | 0.75 | 0.46 | 0.60 | 0.63 | 0.73 | 0.60 | 0.69 | 0.79 | 0.72 | 0.69 | 0.79 | 0.74 | 0.56 | 0.70 |
| RMSE | 0.57 | 0.53 | 0.77 | 0.70 | 0.49 | 0.77 | 0.75 | 0.66 | 0.89 | 0.80 | 0.78 | 0.69 | 0.80 | 0.73 | 0.64 | 0.69 | 0.73 | 0.62 | 0.68 | 0.83 | 0.72 |
