# Supplementary material for: A-to-I RNA editing profiles and distinct immune responses in myelin oligodendrocyte glycoprotein antibody-positive optic neuritis
Source: Front Immunol. 2026 Sep 3;17:1946792. doi: 10.3389/fimmu.2026.1946792 (PMC13582522; doi:10.3389/fimmu.2026.1946792)
Supplement: Supplementary file 3 [file Table5.docx]

**Supplementary Table 5** Concordance between immune cell estimates derived from CIBERSORTx and ImmuCellAI

| **Cell type** | **n** | **Spearman rs** | **95% CI** | **Raw P** | **BH-adjusted P** |
| --- | --- | --- | --- | --- | --- |
| Neutrophils | 21 | 0.89 | 0.74 to 0.95 | ＜0.0001 | ＜0.0001 |
| Monocytes | 21 | 0.01 | -0.43 to 0.45 | 0.95 | 0.95 |
| CD8⁺ T cells | 21 | 0.73 | 0.43 to 0.89 | 0.0002 | 0.0003 |
| NK cells | 21 | 0.66 | 0.31 to 0.85 | 0.001 | 0.001 |

CI: confidence interval; BH: Benjamini–Hochberg; For NK cells, resting and activated NK cell proportions estimated by CIBERSORTx were summed before comparison with ImmuCellAI.
